# Covalent Bridges in Bi Loaded BiVO_4_ Enabling Rapid Charge Transfer for Efficient Photocatalytic Water Oxidation

**DOI:** 10.1002/advs.202500666

**Published:** 2025-06-05

**Authors:** Liyang Li, Zhiming Chen, Dong Fang, Jingxiang Low, Jianhong Yi

**Affiliations:** ^1^ Faculty of Materials Science and Engineering Kunming University of Science and Technology Kunming 650093 P. R. China; ^2^ School of Physical Science and Technology Tiangong University Tianjin 300387 P. R. China

**Keywords:** BiVO_4_, covalent bridge, electron transfer, heterojunction, photocatalysis

## Abstract

Bismuth vanadate (BiVO_4_) is known as one of the most potential candidates in photocatalytic water oxidation for supplying oxygen in extreme environment. However, its photocatalytic oxygen evolution is hindered by the rapid photogenerated charge carrier separation efficiency. Herein, plasmonic bismuth (Bi) nanoparticles loaded BiVO_4_ is prepared for photocatalytic water oxidation. Specifically, the plasmonic bismuth nanoparticles are in situ loaded on the BiVO_4_ via reduction of partial BiVO_4_, allowing the formation of the Bi─O─V covalent bridges. Based on the femtosecond transient absorption spectroscopy and density functional theory calculations, such Bi─O─V covalent bridges can significantly facilitate the migration of the plasmonic‐induced hot electrons from Bi to BiVO_4_, allowing more photogenerated charge carrier to participate in the surface reaction. As a result, the optimized Bi/BiVO_4_ demonstrates a record‐high photocatalytic evolution rate of 4567.94 µmol h^−1^ g^−1^. More importantly, the obtained Bi/BiVO_4_ show plausible photocatalytic water oxidation capability (oxygen production rate of 381.47 µmol h^−1^ g^−1^) under near‐infrared light irradiation, further collaborating its potential to be utilized in extreme conditions. This work on design of low‐cost and highly‐efficient photocatalysts for water oxidation is anticipated to push forward the development of photocatalytic oxygen production in various application scenarios.

## Graphical abstract

Plasmonic bismuth nanoparticles loaded BiVO_4_ photocatalyst is successfully prepared for photocatalytic water oxidation. The in situ loading of the Bi on the BiVO_4_ allows the formation of the Bi–O–V covalent bridges, which can significantly facilitate the photogenerated charge carrier migration accross Bi and BiVO_4_ for enhancing photocatalytic performance.

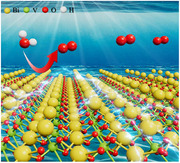

## 1 Introduction

Photocatalytic water oxidation for oxygen production has recently attracted wide attention from the scientific community as it can provide immediate oxygen support in extreme environment, such as high‐altitude areas, underwater, and extraterrestrial sites.^[^ ^1^, ^2^, ^3^ ^]^ In the past several decades, numerous semiconductors, such as TiO_2_,^[^ ^4^, ^5^ ^]^ ZnO^[^ ^6^ ^]^ and WO_3_,^[^ ^7^ ^]^ have been studied for such a reaction. Among them, BiVO_4_ has well‐accepted to be one of the most potential candidates, due to its unique physicochemical properties, such as low bandgap value of 2.4 eV, high oxidation capability, and tunable surface properties.^[^ ^8^, ^9^ ^]^ However, the rapid photogenerated charge carrier recombination and the limited number of active sites on the BiVO_4_ photocatalysts greatly suppressed its photocatalytic performance.^[^ ^10^, ^11^ ^]^ With these problems in mind, a series of modification strategies have been adopted for enhancing the photocatalytic oxygen evolution performance, including metal loading, defect engineering, heterojunction construct, and so on.

Amidst development of the advanced BiVO_4_ materials for photocatalytic oxygen evolution, localized surface plasmon resonance (LSPR) induced by plasmonic metals holds great promise because it can induce near‐field effect for facilitating photogenerated charge carrier separation and extend the light absorption range of the photocatalyst.^[^ ^12^ ^]^ More interestingly, the extended light absorption caused by plasmonic metal can generate hot electrons and holes upon excitation for directly participating in photocatalytic reactions.^[^ ^13^, ^14^ ^]^ For example, *Lee* et al. utilized the plasmonic resonant energy transfer using Au nanoparticles for enhancing the photocatalytic performance of the BiVO_4_.^[^ ^15^ ^]^ Despite the gigantic development in the plasmonic metal‐loaded photocatalysts, the effective migration of the hot electrons from the plasmonic metals to the neighboring semiconductor remains a great challenge. In addition, the current researches of the plasmonic metal for photocatalysis mainly focus on the noble plasmonic metals, especially Au nanoparticles,^[^ ^16^ ^]^ inducing high cost for photocatalyst preparation. Therefore, the development of the low cost plasmonic metals along with effective electron transfer channel from plasmonic metals to semiconductor remains a critical topic in this research field.

Herein, we develop a simple yet effective strategy for in situ loading the non‐noble Bi plasmonic metals on the surface of the BiVO_4_. Specifically, a 34‐faceted BiVO_4_ is first synthesized using a simple one‐pot hydrothermal method, followed by calcination in reductive atmosphere for partially reduced the Bi metal on the surface of the BiVO_4_. More importantly, the in situ transformation of the partial BiVO_4_ to metallic Bi can allow the formation of the Bi─O─V covalent bridge between the metal and semiconductor, providing a rapid charge transfer channel toward facilitating the photogenerated charge carrier migration. As a result, a significant water oxidation rate of 4567.94 µmol h^−1^ g^−1^ can be achieved, which is 2.5 times higher than that of the pristine BiVO_4_. This work not only addresses the slow kinetics of water oxidation in BiVO_4_ but also offers new insights into the rapid transfer of hot electrons.

## 2 Results and Discussion

**Figure 1:**
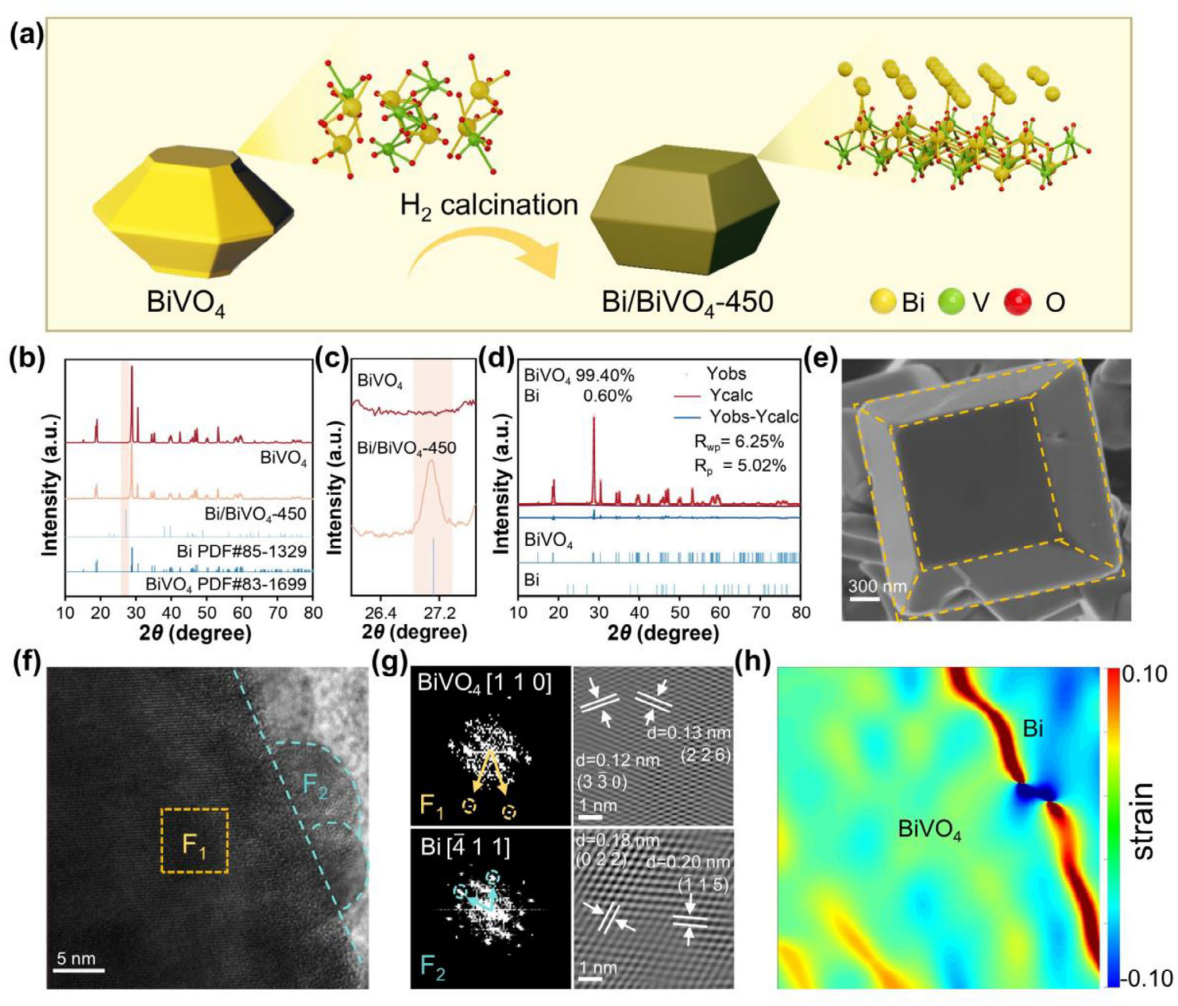
a) Schematic illustration of the preparation procedure of Bi/BiVO_4_‐450from BiVO_4_. b–d) XRD patterns (b), magnified XRD patterns (c) and XRD refinement results of the prepared samples. e) SEM image of Bi/BiVO_4_‐450. f,g) HRTEM (f), corresponding selected area electron diffraction (g) and GPA strain mapping images of Bi/BiVO_4_‐450.

In this work, we aim to in situ generate Bi on the surface of BiVO_4_. To achieve this, BiVO_4_ is calcined in a reducing atmosphere (H_2_ atmosphere calcination), facilitating its partial reduction to metallic Bi (see **Figure** **1**a for the synthesis process). We first study the phase‐structure of the prepared samples via X‐ray diffraction (XRD) characterizations. As shown in Figure 1b, all diffraction peaks of the prepared BiVO_4_ can be indexed to monoclinic BiVO_4_ (JCPDS No. 83–1699). Upon forming metallic Bi on the BiVO_4_, Bi/BiVO_4_‐450 and Bi/BiVO_4_‐500 demonstrates several additional peaks at 27.16, 37.95, and 39.62°, attributed to the (012), (104), and (110) planes of Bi, respectively (see also Figure S1, Supporting Information). It should be noted here that the Bi peaks are absence in Bi/BiVO_4_‐400 because metallic Bi can hardly be formed at a relatively low reaction temperature.^[^ ^17^ ^]^ Moreover, the intensity of Bi diffraction peaks increases with increasing calcination temperature, indicating the increase in crystallization of the formed metallic Bi. Additionally, XRD refinement results shown in Figure 1c, Figure S2 and Table S1 (Supporting Information) indicate that Bi/BiVO_4_‐450 exhibits reduced lattice parameters compared to pure BiVO_4_, suggesting the formation of lattice suppression during the transformation of partial BiVO_4_ into Bi.^[^ ^18^ ^]^ Furthermore, the compositions of BiVO_4_ and Bi in Bi/BiVO_4_‐450 are determined to be ca. 99.40% and 0.60%, respectively.

Microstructural characteristics of Bi/BiVO_4_‐450 and BiVO_4_ were analyzed using scanning electron microscopy (SEM) and high‐resolution transmission electron microscopy (HRTEM). As revealed by the SEM image (Figure S3a, Supporting Information), the prepared BiVO_4_ exhibits a significant 34‐faceted morphology, particularly with its top and bottom surfaces showing distinct octagonal structures. After reduction of BiVO_4_ at 450 °C in H_2_, the obtained Bi/BiVO_4_‐450 exhibits a distinct decahedral morphology (see Figure 1e; Figure S4, Supporting Information). According to Bravais's law, the crystal morphology is closely related to the orientation of its crystal planes. In this study, the emergence of the decahedral morphology is attributed to the preferential orientation of (010) and (110) crystal planes during the sintering process. This indicates that during high‐temperature reduction treatment, certain specific crystal planes exhibit a higher energy effect, leading to their preferential orientation during growth. Simultaneously, to minimize the total surface energy, other high‐angle crystal planes disappears during the crystal growth process, resulting in the decahedral morphology observed in Bi/BiVO_4_‐450. Moreover, compared to Bi/BiVO_4_‐400 and Bi/BiVO_4_‐500, the surface of the Bi/BiVO_4_‐450 exhibits a more regular morphology. Notably, the morphological transformation cannot be observed over the Bi/BiVO_4_‐400 (Figure S3b, Supporting Information), suggesting that the relatively low temperature can hardly trigger the morphological transformation of the BiVO_4_. In addition, the surface of the decahedral Bi/BiVO_4_‐500 becomes uneven due to the destruction of the surface structure of the BiVO_4_ (Figure S3c, Supporting Information). The inductively coupled plasma (ICP) analysis reveals that the vanadium (V) content in Bi/BiVO_4_‐500 is lower than that in Bi/BiVO_4_‐450 (Table S2, Supporting Information), implying the loss of the V at elevated temperatures.

Furthermore, the heterojunction structure of the Bi/BiVO_4_ can be confirmed via the HRTEM characterizations (Figure 1f,g). Specifically, lattice fringes with spacings of 0.12 and 0.13 nm can be observed in the F_1_ region, corresponding to the (3$\bar{3}$0) and (2$\bar{2}$6) planes of monoclinic BiVO_4_ along the [110] axis, respectively. In the F_2_ region, lattice fringes with spacings of 0.18 and 0.20 nm are indexed to the ($02\bar{2}$) and ($\bar{1}\bar{1}5$) planes of the hexagonal Bi along the [$\bar{4}11$] axis. This further confirms the intimate integration of Bi nanoparticles with BiVO_4_, which enhances the electronic interactions between Bi and BiVO_4_. The energy dispersive X‐ray spectroscopy (EDS) mapping images show a uniform distribution of Bi, V, and O elements (Figure S5 and Table S3, Supporting Information). The electron energy loss spectroscopy (EELS) analysis shown in Figure S6 (Supporting Information) further reveals the binary nature of BiVO_4_ and Bi within Bi/BiVO_4_‐450. The geometric phase analysis (GPA) is employed to study the dislocation and strain distribution.^[^ ^19^ ^]^ As shown in Figure 1h and Figure S7 (Supporting Information), Bi/BiVO_4_‐450 exhibits more strain and defects, primarily concentrated at the interface between Bi and BiVO_4_, compared to BiVO_4_, endowing more active sites for the subsequent photocatalytic reaction.

**Figure 2:**
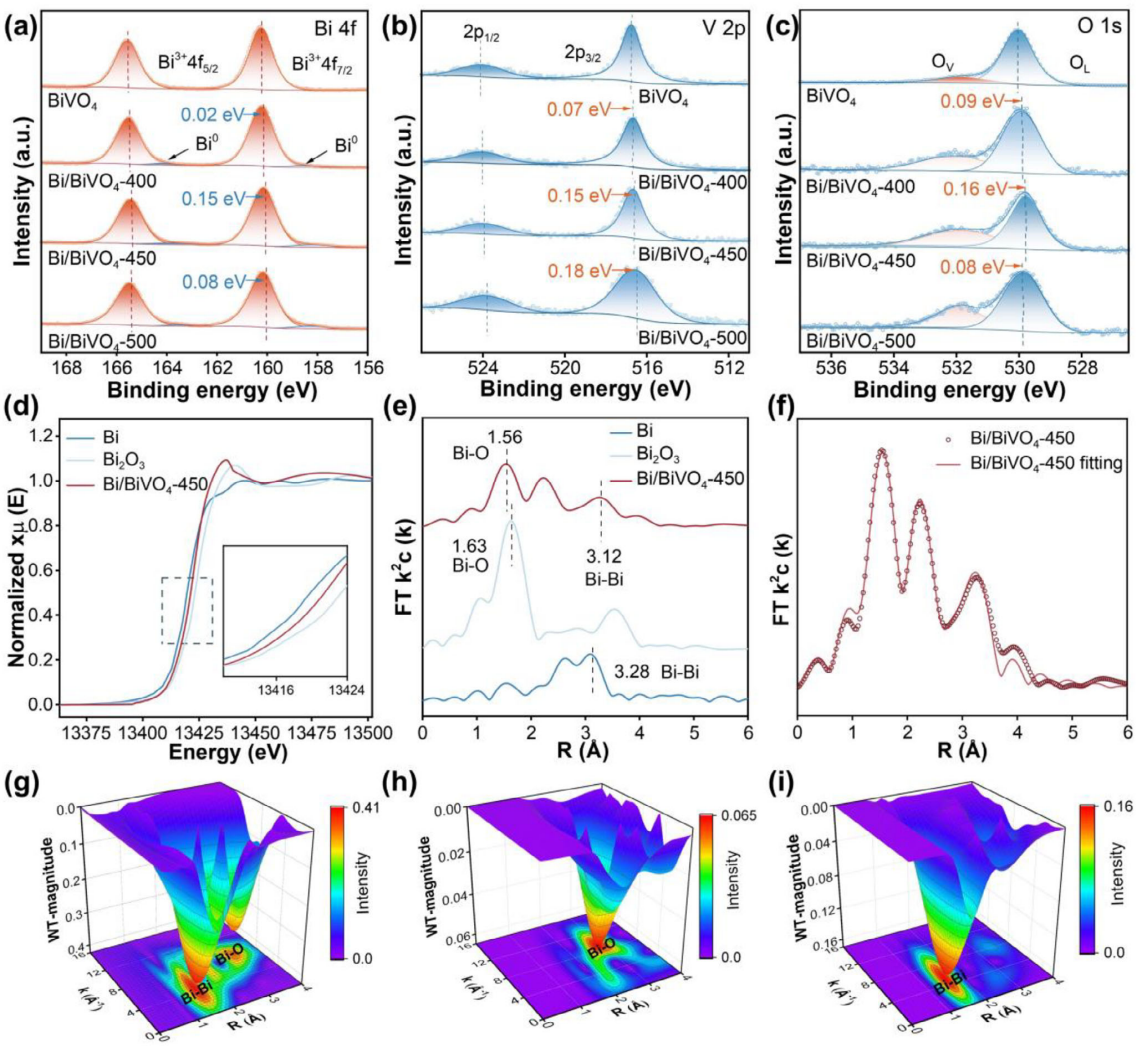
a–c) High resolution V 2p (a), Bi 4f (b) and O 1s (c) XPS spectra of the prepared samples. d) Normalized Bi K‐edge XANES spectra of different samples. e) Radial distance χ(R) space spectra at the Bi K‐edge of different samples. f) Fitting curves of Fourier transform (FT) extended X‐ray absorption fine structure (EXAFS) at the Bi K‐edge R space of Bi/BiVO_4_‐450. g–i) 3D wavelet transform plots at the Bi K‐edge for Bi (g), Bi_2_O_3_ (h) and Bi/BiVO_4_‐450 (i).

To evaluate the chemical status of the prepared samples, X‐ray photoelectron spectroscopy (XPS) was employed. The XPS survey spectra of the prepared samples confirm the coexistence of Bi, O, and V elements (**Figure** **2**a–c; Figure S8, Supporting Information), which is in consistent with the EDS results. For the BiVO_4_, its high‐resolution Bi 4f XPS spectrum demonstrates two peaks at 159 and 164 eV, attributed to Bi 4f_5/2_ and Bi 4f_7/2_ of BiVO_4_, respectively (Figure 2a).^[^ ^20^, ^21^ ^]^ In addition, two peaks at 516 and 524 eV can be observed in the high‐resolution V 2p XPS spectrum of BiVO_4_, corresponding to V 2p_3/2_ and V 2p_1/2_ of V^5+^, respectively (Figure 2b).^[^ ^22^, ^23^ ^]^ Compared to BiVO_4_, the binding energy peak positions of the Bi 4f, V 2p and O 1s over Bi/BiVO_4_‐400, Bi/BiVO_4_‐450, and Bi/BiVO_4_‐500 significantly shift to a more negative binding energy values, implying the increase in the electron density of BiVO_4_ over these samples.^[^ ^24^, ^25^ ^]^ Notably, two additional peaks at 157 and 163 eV, corresponding to the Bi^0^, can be observed in the high‐resolution Bi 4f XPS spectra of these samples, suggesting the presence of the metallic Bi.^[^ ^26^ ^]^ Interestingly, no metallic V can be observed in the samples because the unsaturated Bi in the BiVO_4_ is more easily to be reduced compared to the V element in BiVO_4_.^[^ ^27^ ^]^ Additionally, the formation of covalent bonds alters the electronic states of the atoms, affecting the peak positions and shapes in the XPS spectra. As shown in Figure 2c, compared to BiVO_4_, the differences in peak positions and areas of the O 1s XPS spectra in Bi/BiVO_4_ indicate that the coordination environment of O atoms changes due to the loading of metallic Bi. This further confirms the presence of strong interactions between Bi and the BiVO_4_ matrix. It should be noted here that the formation of the metallic Bi is accompanied by the emergence of the oxygen vacancies (O_V_, see Figure 2c). The proportion of O_V_ in Bi/BiVO_4_‐450 (30.51%) is higher than those in other prepared samples (Table S4, Supporting Information).^[^ ^17^ ^]^ As revealed by the ESR characterization in Figure S9 (Supporting Information), significant peak can be observed at a g‐value of 2.002, corresponding to the Zeeman effect of unpaired electrons captured by O_V_.^[^ ^21^, ^28^ ^]^ In addition, the intensity of the peak over Bi/BiVO_4_‐450 is significantly higher than that over BiVO_4_ under both visible light and dark conditions, indicating a higher concentration of O_V_ in Bi/BiVO_4_‐450.

To further elucidate the electronic structure and coordination environment of Bi in Bi/BiVO_4_‐450, the X‐ray absorption fine structure (XAFS) spectroscopy was performed. The Bi K‐edge X‐ray absorption near edge structure (XANES) spectra of Bi foil, Bi_2_O_3,_ and Bi/BiVO_4_‐450 are shown in Figure 2d. Generally, the Bi absorption edge of Bi/BiVO_4_‐450 lies between those of Bi foil and Bi_2_O_3_. This observation indicates that the oxidation state of Bi in the sample ranges from 0 to +3. The Fourier transform *k* ^3^‐weighted extended X‐ray absorption fine structure (FT‐EXAFS) spectra of different samples are presented in Figure 2e and Figure S10a (Supporting Information). The peak at 1.56 Å in Bi/BiVO_4_‐450 corresponds to the Bi─O bond, while the peak at 3.12 Å is associated with the Bi─Bi bond. Quantitative fittings of the data in both *k* space and R space (Figure 2f; Figure S10b–d, Supporting Information) reveal that Bi/BiVO_4_‐450 contains Bi─Bi and Bi─O bonds. The Bi─O and Bi─V/Bi signals are attributed to the scattering from BiVO_4,_ whereas the Bi─Bi signal corresponds to metallic Bi, further confirming the coexistence of BiVO_4_ and Bi (Table S5, Supporting Information). The wavelet transforms (WT) results shown in Figure 2g–i illustrate the relationship between the *k* space and R space in the samples. The peak at 4 Å^−1^ in Bi/BiVO_4_‐450 aligns with the Bi─Bi bond in elemental Bi, while the peak at 7.9 Å^−1^ corresponds to the Bi─O bond in Bi_2_O_3_.

To evaluate the surface active sites on the prepared samples, the nitrogen adsorption‐desorption isotherms of the prepared samples were obtained. Typically, all the prepared samples demonstrate a type‐IV pattern (Figure S11a, Supporting Information), indicating their mesoporous structure. The non‐closure feature of the BiVO_4_ isotherm is attributed to its small specific surface area.^[^ ^29^, ^30^ ^]^ The specific surface areas of BiVO_4_ and Bi/BiVO_4_‐450 are determined to be 0.46 and 0.97 m^2^ g^−1^, respectively, suggesting that the reduction of Bi^3+^ to Bi^0^ during synthesis generates additional pores in the material. The pore sizes of the samples range from 2 to 10 nm (Figure S11b, Supporting Information), indicating their mesoporous structure. These mesopores provide efficient transport pathways for the reactants and products during photocatalytic reactions.^[^ ^29^ ^]^

### 2.1 Photoelectrochemical Properties and Photocatalytic Performance

**Figure 3:**
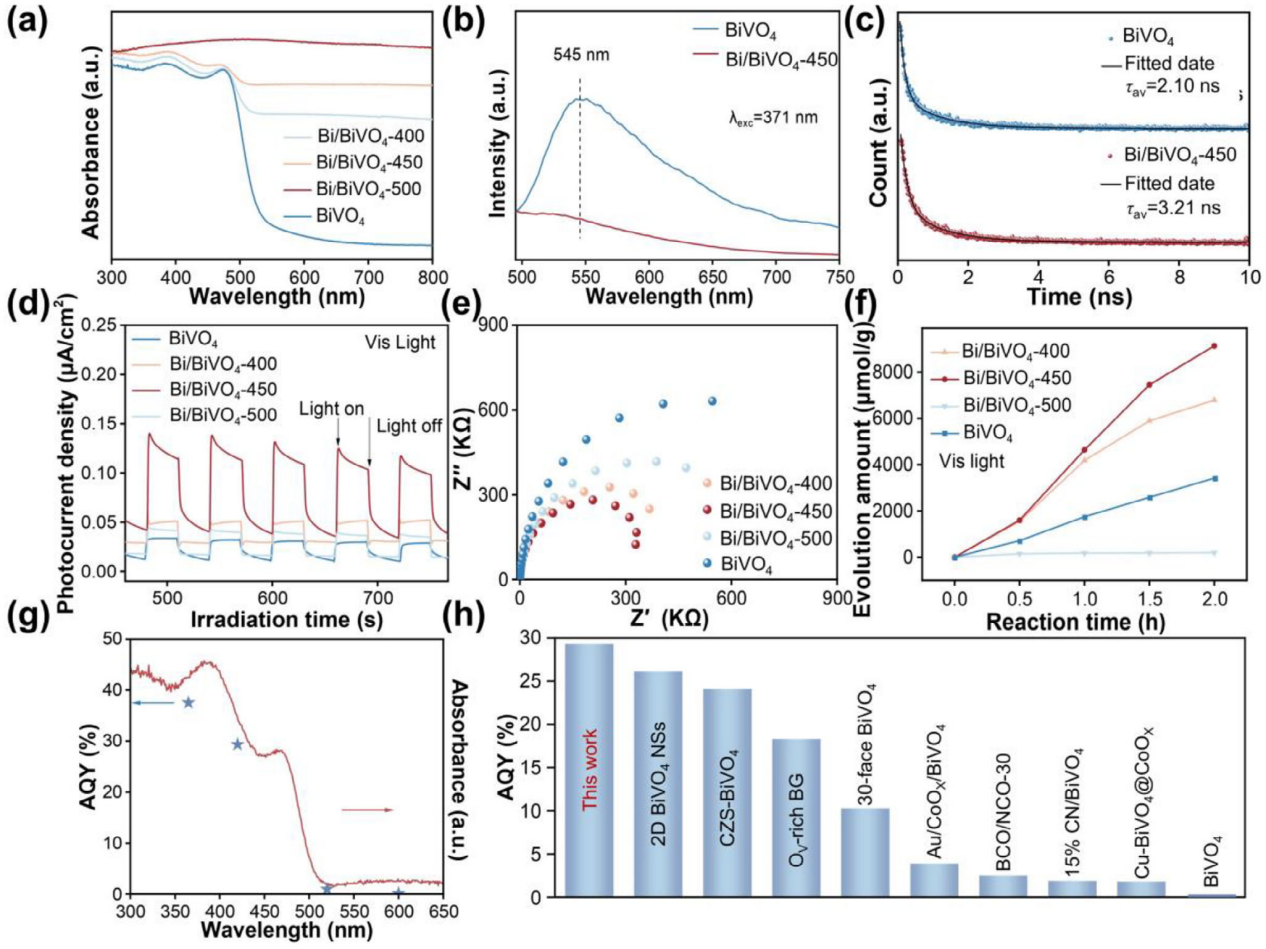
a) UV–vis absorption spectra of the prepared samples. b,c) PL emission spectra (b) and TRPL decay spectra (c) of BiVO_4_ and Bi/BiVO_4_‐450. d,e) Electrochemical transient photocurrent responses spectra (d) and Nyquist plot (e) of the prepared samples. f) Photocatalytic O_2_ production of the prepared samples under visible light (λ > 420 nm) irradiation. g) Wavelength‐dependent AQY for photocatalytic oxygen evolution over Bi/BiVO_4_‐450 at different wavelength. h) Comparison of AQY for photocatalytic oxygen evolution of Bi/BiVO_4_‐450 with recently reported photocatalysts.

The light‐responsive capability of the prepared samples was studied via the (UV–vis) diffuse reflection absorption spectroscopy and the results are shown in **Figure** **3**a. Generally, Bi/BiVO_4_‐400, Bi/BiVO_4_‐450, and Bi/BiVO_4_‐500 exhibit enhanced absorption intensity in the visible and near‐infrared regions compared to BiVO_4_, accompanied by a redshift in the absorption edge. This expansion in the range and intensity of light absorption enables the photocatalyst to capture more photons for subsequent chemical reactions. The equation $\left(\alpha h\upsilon )n=A(h\upsilon \;-\;E_{g}\right)$ is used to calculate the band gaps (*E_g_*) of the prepared samples.^[^ ^30^, ^31^, ^32^ ^]^ Based on this calculation, the bandgap values of BiVO_4_ and Bi/BiVO_4_‐450 are determined to be ca. 2.47 and 1.96 eV, respectively (Figure S12, Supporting Information).

Furthermore, the photoluminescence (PL) characteristics of the catalysts were examined to elucidate the charge separation efficiency of the prepared samples (Figure 3b).^[^ ^33^, ^34^ ^]^ BiVO_4_ displays a pronounced emission peak at 545 nm, attributed to the extensive recombination of photogenerated charge carriers. In contrast, Bi/BiVO_4_‐450 exhibits a substantial reduction in PL peak intensity, suggesting its suppressed photogenerated charge carrier recombination compared to the BiVO_4_.^[^ ^35^ ^]^ To quantitatively investigate the photogenerated charge carrier lifetimes of the prepared samples, time‐resolved photoluminescence (TRPL) spectra are obtained (Figure 3c), and the corresponding decay curves are fitted using a double‐exponential model. Specifically, the increase in fluorescence lifetime indicates enhanced photogenerated charge carrier separation efficiency and a reduced recombination rate of charge carriers over the studied samples.^[^ ^36^, ^37^, ^38^ ^]^ BiVO_4_ exhibits an average carrier lifetime (τ_av_) of 2.10 ns, while Bi/BiVO_4_‐450 shows a significantly extended lifetime of 3.21 ns. This finding underscores the superior photogenerated charge separation efficiency of Bi/BiVO_4_‐450 compared to other studied samples.^[^ ^37^ ^]^ This enhanced charge carrier separation efficiency is because the in situ transformation of the partial BiVO_4_ into Bi can allow the formation of the Bi─O─V covalent bridges, thereby facilitating the photogenerated charge carrier migration efficiency of the Bi/BiVO_4_‐450. In addition, the defect energy levels induced by O_V_ can also influence the photo‐relaxation process. Specifically, photogenerated electrons from the VB of BiVO_4_ can transfer to the defect energy levels via radiative decay, thereby prolonging τ_av_.^[^ ^39^ ^]^

To further investigate photogenerated charge carrier dynamics, photoelectrochemical testing was conducted. Under light irradiation, the photocurrent density of Bi/BiVO_4_‐450 reaches 0.12 µA cm^−2^, which is at least ca. three times higher than those of the other studied samples (Figure 3d; Figure S13a, Supporting Information). Additionally, Bi/BiVO_4_‐450 demonstrates a higher response than BiVO_4_ under near‐infrared light irradiation, indicating that the incorporation of Bi broadens the spectral response range into the near‐infrared region (Figure S13c, Supporting Information). Furthermore, the photocurrent remains stable over five on/off light cycles without significant deterioration, demonstrating that the Bi/BiVO_4_‐450 owns a high photostability.^[^ ^40^ ^]^ In addition, the electrochemical impedance spectroscopy (EIS) spectra reveal that, compared to other studied samples, Bi/BiVO_4_‐450 exhibits a smaller radius (Figure 3e; Figure S13b, Supporting Information), indicating reduced impedance to charge transfer and enhanced interfacial electron mobility.^[^ ^41^, ^42^ ^]^ These electrochemical results demonstrate that Bi/BiVO_4_‐450 offers higher charge separation efficiency and faster internal electron transfer compared to the other samples.

Upon having a comprehensive understanding on the physicochemical properties of the prepared samples, the photocatalytic oxygen evolution performances of the prepared samples are evaluated (Figure 3f; Figures S14 and S15a, Supporting Information). Notably, among all the prepared samples, Bi/BiVO_4_‐450 exhibits the highest photocatalytic oxygen evolution rate, achieving 4567.94 µmol h^−1^ g^−1^ after 2 h of visible‐light irradiation, exceeding the performance of pristine BiVO_4_ for ca. 2.5 times. Moreover, such a performance can be maintained for up to five cycles of reaction, further confirming its high stability (Figure S15b, Supporting Information). The poor performance of Bi/BiVO_4_‐500 is mainly due to vanadium loss at high temperature, which results in the collapse of the tetrahedral structure and disruption of electron transfer pathways.^[^ ^42^ ^]^ Furthermore, ICP testing is also conducted on the Bi/BiVO_4_‐450 after the reaction. Interestingly, negligible changes in V content can be observed on the Bi/BiVO_4_‐450 before and after reaction (Table S2, Supporting Information), further confirming its high stability. To further demonstrate the superiority of the prepared Bi/BiVO_4_‐450, the photocatalytic oxygen evolution performance of the Bi/BiVO_4_‐450 is compared with that of BiVO_4_ loaded with different precious metals, including Pt and Au (see Figure S16, Supporting Information for their SEM and EDS images). Interestingly, the photocatalytic oxygen evolution performance of Bi/BiVO_4_‐450 is significantly higher than those of the Pt/BiVO_4_ and Au/BiVO_4_ (Figure S15c, Supporting Information), demonstrating the effectiveness of non‐precious metal Bi loading. Moreover, the effect of different catalyst amounts on oxygen evolution performance was evaluated under identical conditions (Figure S15d,e, Supporting Information), revealing that a catalyst amount of 50 mg achieves the optimal performance. More importantly, the AQY of Bi/BiVO_4_‐450 is 29.32% at 420 nm (Figure 3g), which is one of the highest among recently reported photocatalysts (Figure 3h; Table S6, Supporting Information), indicating its outstanding photocatalytic efficiency.

To further demonstrate the roles of the Bi─O─V covalent bridge in enhancing photocatalytic performance, we introduced metallic Bi onto BiVO_4_ via a chemical reduction method to avoid the formation of the Bi─O─V covalent bridge (see Experimental Section in Supporting Information). As shown in Figure S17 (Supporting Information), the XRD characterization reveal the presence of metallic Bi and BiVO_4_ on the Bi/BiVO_4_‐CR. In addition, Figure S15f (Supporting Information) shows that the photocatalytic oxygen evolution performance of the Bi/BiVO_4_‐CR is significantly lower than that of the Bi/BiVO_4_‐450, suggesting that the absence of the Bi─O─V covalent bridge can suppress the photogenerated charge carrier migration transfer across the Bi and BiVO_4_ interface. Notably, the oxygen evolution rate for Bi/BiVO_4_‐450 reaches 381.47 µmol h^−1^ g^−1^ under near‐infrared light irradiation (Figure S18, Supporting Information), suggesting its capability of utilizing a whole spectrum of incident solar energy. This result suggests that the presence of the metallic Bi cannot only enhance the photocatalytic performance of the BiVO_4_, but also extend its light‐responsive range into the near‐infrared light range.

### 2.2 Ultrafast Plasmon‐Induced Charge Transfer and Photocatalytic Mechanism

**Figure 4:**
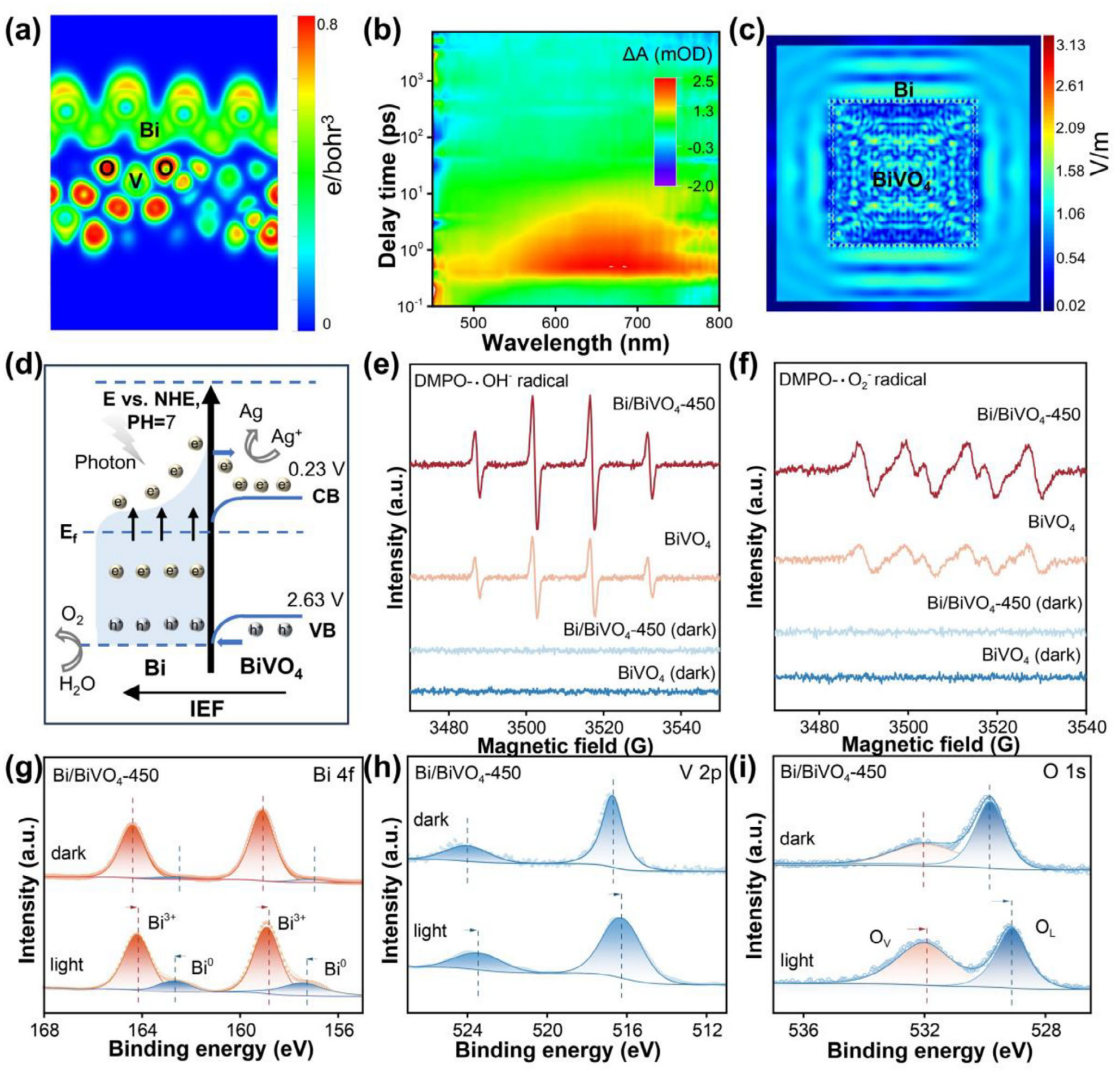
a) Electron localization function (ELF) plot of Bi/BiVO_4_‐450. b) 2D pseudo‐color plot of fs‐TA spectra of Bi/BiVO_4_‐450 under irradiation of 380 nm fs laser pulses. c) Distribution of the strongest locally enhanced electric field on the X‐Y plane of Bi/BiVO_4_‐450 under visible light irradiation with a wavelength of 420 nm. d) Schematic illustration of photogenerated charge carrier migration over Bi/BiVO_4_‐450. e,f) ESR signals of •O_2_^−^ (e) and •OH (f) radicals under visible light (λ > 420 nm) and in dark conditions. g–i) In situ light‐irradiated high‐resolution Bi 4f (g), V 2p (h) and O 1s (i) XPS spectra of Bi/BiVO_4_‐500.

The electron localization function (ELF) of Bi/BiVO_4_‐450 was also calculated by DFT. Structural diagram reveals the presence of shared electron pairs and strong metal‐substrate interactions between Bi and BiVO_4_ (**Figure** **4**a; Figure S19, Supporting Information). It is revealed that there are shared electron pairs between metallic Bi and the VO tetrahedra in BiVO_4_ to form the Bi─O─V covalent bridge, consistent with the results from HRTEM and XPS.^[^ ^17^ ^]^ The role of the Bi─O─V covalent bridge of Bi/BiVO_4_‐450 in accelerating electron transport is further elucidated using femtosecond transient absorption (fs‐TA) spectroscopy. Under 380 nm light excitation, the fs‐TA of Bi exhibits a ground‐state bleaching signal at 525 nm (Figures S20a and S21d, Supporting Information), which is induced by the plasmon resonance of Bi.^[^ ^43^ ^]^ For the BiVO_4_ and Bi/BiVO_4_‐450 (Figure 4b; Figure S20b, Supporting Information), the positive signal in the 490–700 nm range is observed, corresponding to the excited‐state absorption (ESA) processes of electron‐hole pairs.^[^ ^44^ ^]^ In addition, the intensity of the ESA signal is directly associated with the dynamics of carrier separation and transport.^[^ ^45^ ^]^ Therefore, we compare the ESA signal of all the prepared samples. The fs‐TA spectrum of Bi/BiVO_4_‐450 displays a stronger signal intensity compared to the BiVO_4_, suggesting a higher number of electrons on Bi/BiVO_4_‐450 and extended charge relaxation times. Since the energy of the excited plasmonic excitons (380 nm) is substantially lower than the absorption onset wavelength of BiVO_4_ (ca. 500 nm), the LSPR band of Bi in Bi/BiVO_4_‐450 is selectively excited.^[^ ^19^, ^46^ ^]^ Based on these characterizations, it can be concluded that the plasmonic‐induced hot electrons from Bi are effectively transferred to BiVO_4_. Meanwhile, the fs‐TA spectra reveal that the absorption of both BiVO_4_ and Bi/BiVO_4_‐450 gradually decreases with increasing probe delay times, indicating a continuous attenuation of absorption (Figure S21b,c, Supporting Information). This trend suggests that the absorption of both samples diminishes over time. Therefore, it can confirmed that Bi promotes charge transfer and functions as a covalent bridge, facilitating the rapid transfer of hot electrons across the interface between Bi and BiVO_4_.^[^ ^45^ ^]^ To investigate the decay dynamics of ESA, the fs‐TA decay trajectories of BiVO_4_ and Bi/BiVO_4_‐450 at specific wavelengths (538 and 650 nm) are fitted. The resulting time constants (*τ*_1_, *τ*_2_ and *τ_av_*) are summarized in Figure S22 and Table S7 (Supporting Information). Compared to BiVO_4_, Bi/BiVO_4_‐450 exhibits longer charge carrier recombination lifetime (*τ*_1_) and extended shallow charge trapping lifetimes (*τ*_2_). These results confirm the persistence of the excited‐state absorption, allowing more photogenerated electrons to participate in the surface reaction.^[^ ^47^, ^48^ ^]^ In addition, the finite‐difference time‐domain (FDTD) simulations further corroborate the enhancement of the electric field and LSPR effects of Bi. The 3D model and cross‐sectional view of Bi/BiVO_4_‐450 are shown in Figure S23a,b (Supporting Information). Notably, localized “hot spots” are observed between the Bi (see Figure 4c), indicating intensified electric field at the Bi/BiVO_4_‐450 interface compared to incident electromagnetic radiation. This enhancement facilitates the separation of photogenerated electron‐hole pairs near the Bi/BiVO_4_‐450 surface.^[^ ^49^, ^50^ ^]^

The valence band position of the samples was further determined through ultraviolet photoelectron spectroscopy (UPS). The cutoff energies of BiVO_4_ and Bi/BiVO_4_‐450 are calculated to be 17.90 and 17.49 eV, respectively (Figure S24a,b, Supporting Information). The valence band edge (E_VB_) of BiVO_4_ is determined to be 7.09 eV using the equation: *EVB*  =  *h*ν  − (*E_cutoff_*  − *E_onset_*), where the incident photon energy *hν* is 21.22 eV.^[^ ^34^ ^]^ Based on this result, the conduction band edge (E_CB_) of the BiVO_4_ can be estimated to be 4.62 eV based on the E_VB_ and *E_g_* calculations. Moreover, the E_VB_ and E_CB_ values can be determined to be 2.63 and 0.23 V, respectively, according to the reference standard (0 V vs the reversible hydrogen electrode corresponds to −4.44 eV). Additionally, the *Φ* of BiVO_4_ and Bi/BiVO_4_‐450 are calculated to be 4.71 and 4.68 eV, respectively, using the equation of Φ  =  *h*ν  − |*E_cutoff_*  − *E_f_* |, where the incident photon energy *hν* set at 21.22 eV, and *E_f_* calibrated to 0 eV using a standard Au sample.^[^ ^51^ ^]^ Notably, the Fermi levels of BiVO_4_ and Bi are 4.71 and 4.20 eV, respectively. After the formation of the heterojunction, the Fermi level of Bi/BiVO_4_‐450 tends to align, exhibiting a value of 4.68 eV. The slight Fermi level shift (*ca*. 0.03 eV) in Bi/BiVO_4_‐450 compared to BiVO_4_ can be observed, indicating the formation of a Schottky barrier at the interface between Bi and BiVO_4_.

The reasons behind the excellent photocatalytic performance of Bi/BiVO_4_‐450 are further explored from the perspective of the oxygen evolution mechanism. Upon the formation of the composite, the interface electronic structure between BiVO_4_ and Bi is depicted in Figure S24c (Supporting Information), where the Fermi levels of BiVO_4_ and Bi align to establish a Schottky junction. Upon light irradiation, photon excitation of plasmonic Bi generates hot electrons and hot holes, with the Schottky barrier acting as a selective channel, enabling hot electrons to cross the interface and migrate to the conduction band of the semiconductor (Figure 4d). This mechanism effectively inhibits the reverse flow of hot electrons, enhancing charge separation efficiency and facilitating directional charge transport. As a result, electron‐hole pairs separate at the interface, with the Schottky heterojunction guiding the migration of hot electrons to BiVO_4_, thereby enhancing electron‐hole pair separation and notably elevating the photocatalytic performance under visible light conditions. Furthermore, the Bi─O─V covalent bridge connecting the metal and semiconductor establishes an electron transfer pathway, enabling efficient and rapid injection of hot electrons into BiVO_4_. These high‐energy hot electrons incessantly react with the surface oxygen atoms of BiVO_4_ bonded to Bi, resulting in the formation of O_V_.^[^ ^17^, ^52^ ^]^ This is consistent with the significant increase in O_V_ observed in Bi/BiVO_4_‐450 upon light exposure (see Figure S9, Supporting Information). The O_V_ at the interface acts as an electron trap, capturing electrons and acting as active centers that promote the generation of the oxygen.^[^ ^21^ ^]^

The radicals trapping tests were also performed using 5,5‐dimethylpyrroline N‐oxide (DMPO) as a trapping agent via the ESR characterizations to trace the production of superoxide radicals (•O_2_^−^) and hydroxyl radicals (•OH).^[^ ^36^, ^53^ ^]^ As shown in Figure 4e,f, all three samples exhibit DMPO‐•O_2_^−^ adduct signals and DMPO‐•OH peaks, corresponding to the reduction of O_2_ and the oxidation of H_2_O, respectively. It is noteworthy that Bi/BiVO_4_‐450 exhibits a more intense DMPO adduct signal than BiVO_4_, indicating enhanced molecular oxygen activation. Additionally, the DMPO‐•OH signal is stronger than the DMPO‐•O_2_^−^ signal in pure BiVO_4_ (Figure S25, Supporting Information), suggesting the strong oxidation capability of the BiVO_4_.^[^ ^12^ ^]^ Based on the radicals trapping test, it is obvious that the presence of the Bi can facilitate the photogenerated charge carrier separation toward enhancing the surface redox reaction of the BiVO_4_. In situ light‐irradiated XPS (ISI‐XPS) analysis was also performed to further elucidate the photogenerated charge transfer pathway. As shown in Figure 4g–i, the binding energy peaks attributed to BiVO_4_ of Bi/BiVO_4_‐450 shift toward negative binding energy value after light irradiation, indicating an increase in electron density. In contrast, the binding energy peak attributed to the Bi^0^ shift toward a positive value, implying a reduction in its electron density. This opposite tendency of binding energy shift for Bi and BiVO_4_ indicates the migration of the photogenerated electrons from Bi (i.e., hot electrons) to BiVO_4_ under light irradiation. In addition, as shown in Figure 4i, the proportion of O_V_ increases significantly after illumination, further confirming that the increase in O_V_ is induced by the impact of high‐energy hot electrons from Bi.

### 2.3 DFT Calculation and Analysis

**Figure 5:**
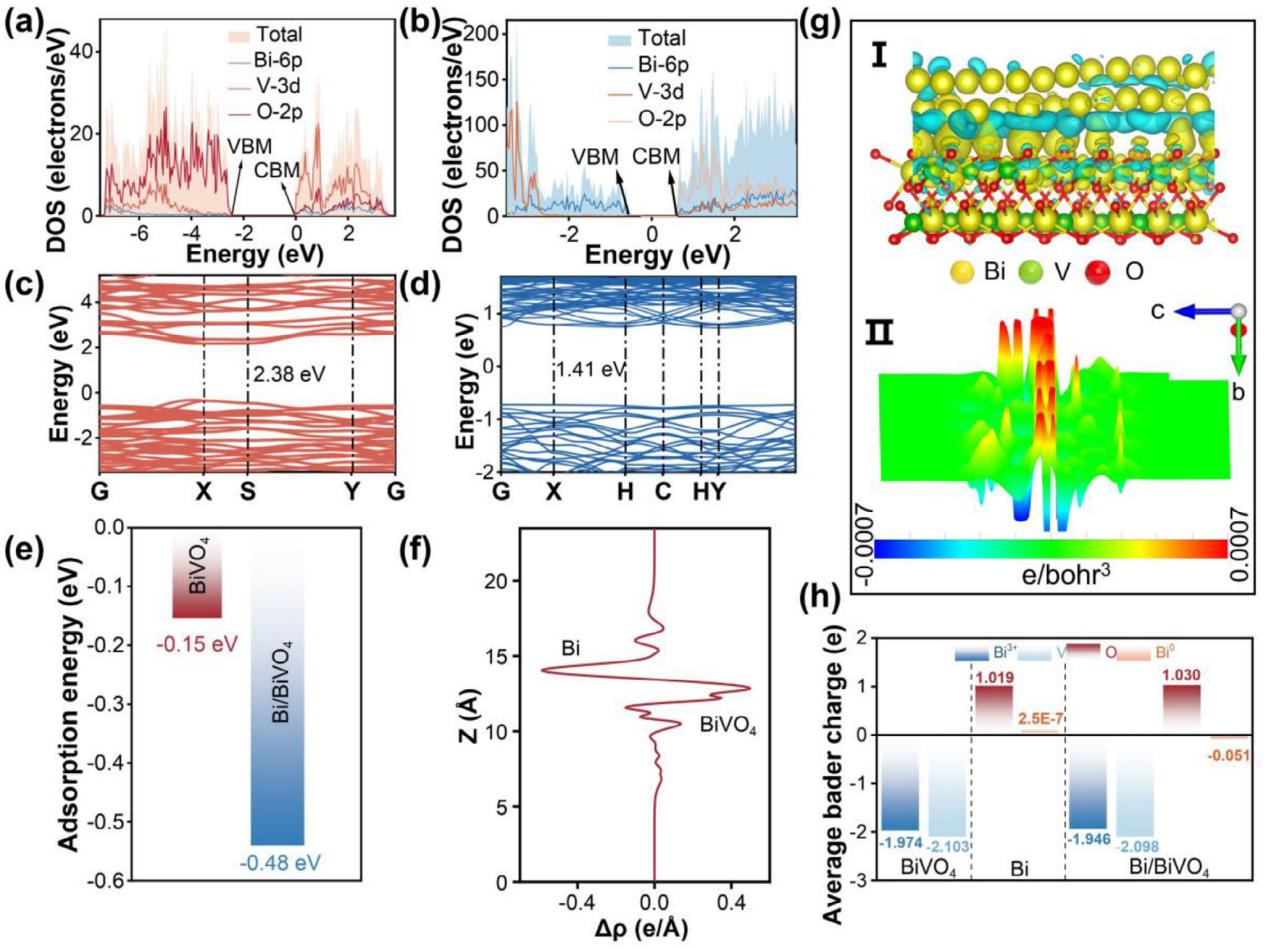
a,b) Total and partial DOS of BiVO_4_ (a) and Bi/BiVO_4_ (b). c,d) Band structures of BiVO_4_ (c) and Bi/BiVO_4_ (d). e) Adsorption energy graphs for BiVO_4_ and Bi/BiVO_4_. f,g) Potential difference diagram and (g) iso‐surfaces of charge‐density difference of Bi/BiVO_4_. h) Bader charge analysis of Bi, BiVO_4_ and Bi/BiVO_4_.

Theoretical calculations provide deeper insights into the hot electron transfer mechanism and charge carrier migration process. BiVO_4_ and Bi/BiVO_4_ models containing O_V_ were constructed and shown in Figure S26 (Supporting Information). The calculated density of states (DOS) and density of states (PDOS) were obtained using these models and the corresponding results are presented in **Figure** **5**a,b. For the BiVO_4_, its VB edge is primarily composed of O 2p orbitals, while the CB edge predominantly consists of V 3d orbitals. For Bi/BiVO_4_, the VB edge shifts toward Bi 6p orbitals, whereas the CB edge is dominated by V 3d orbitals. Compared with BiVO_4_, Bi/BiVO_4_ exhibits a noticeable decrease in the DOS for O atoms. In addition, a peak associated with Bi emerges at a similar position, occupying the VB edge. This indicates that the covalent electrons of Bi are approaching the p orbitals of O from Bi, further confirming the role of Bi as a covalent bridge linking the metal and the carrier, providing an efficient channel for rapid electron transfer from Bi to BiVO_4_. It is well‐known that the Fermi level difference on the difference surfaces of the BiVO_4_ can guide the photogenerated electrons and holes to accumulate on the (010) and (110) surfaces, respectively. To collaborate this, the density of states (DOS) and work function for the (010) and (110) faces are estimated via DFT calculations. Typically, compared to the (010) surface, the (110) surface exhibits higher conduction band (CB) and valence band (VB) levels (Figure S27a,b, Supporting Information) and lower work function (Figure S27c,d, Supporting Information). As such, the photogenerated electrons on the (110) surface tend to migrate to the (010) surface to reach the Fermi equilibrium, resulting in the spatial separation of photogenerated electrons and holes (Figure S28a,b, Supporting Information).

Moreover, the band structures of BiVO_4_ and Bi/BiVO_4_ were also calculated. As shown in Figure 5c,d, theoretical band gap values for BiVO_4_ and Bi/BiVO_4_ are calculated to be 2.38 and 1.41 eV, respectively. The lower calculated value for Bi/BiVO_4_ is attributed to the underestimation of the band gap by the PBE+U method.^[^ ^54^, ^55^ ^]^ It should be noted here that the observed decrease in band gap value of the Bi/BiVO_4_ is also consistent with experimental findings. Specifically, Bi/BiVO_4_ exhibits additional electron‐occupied Bi 6p orbitals. The presence of these additional orbitals leads to a narrowing of the band gap in the Bi/BiVO_4_, thus confirming that the incorporation of Bi reduces the band gap value of the BiVO_4_. Apart from the band structure, the binding of H_2_O on the catalyst surface marks the starting point of the oxygen evolution reaction.^[^ ^56^ ^]^ Therefore, the adsorption energy of H_2_O is another critical parameter for evaluating the performance of the catalyst. The optimized configurations and energetically most favorable structures of H_2_O adsorbed on the surfaces of the BiVO_4_ and Bi/BiVO_4_ are shown in Figure 5e and Figure S29 (Supporting Information). The adsorption energies of H_2_O on BiVO_4_ and Bi/BiVO_4_ are determined to be −0.15 and −0.48 eV, respectively. The negative adsorption energies indicate that the H_2_O adsorption is a spontaneous process. Moreover, the adsorption energy of H_2_O on Bi/BiVO_4_ is significantly lower than on BiVO_4_, suggesting that H_2_O is more easily adsorbed on the Bi/BiVO_4_ surface. Furthermore, the charge distribution between Bi and BiVO_4_ is analyzed to investigate potential pathways for electron transfer. Figure 5g and Figure S30 (Supporting Information) present the calculated spin‐polarized charge density distributions for the Bi/BiVO_4_ crystal containing O_V_. In these plots, yellow and red regions indicate electron accumulation, while cyan and blue regions correspond to electron depletion.^[^ ^57^, ^58^ ^]^ As revealed by these figures, the cyan and blue regions are found to locate on metallic Bi, suggesting the electron depletion behavior of Bi. In addition, the potential difference diagram reveals that Bi exhibits a more negative potential than BiVO_4_ (Figure 5f), suggesting that Bi acts as a charge donor to BiVO_4_. To further investigate the electron transfer process, the Bader charge calculation was conducted on the BiVO_4_ (Figure 5h), Bi and Bi/BiVO_4_ systems. The calculation clearly shows that Bi loses 0.05 |e|, indicating a decrease in charge density around Bi, which corresponds to the differential charge results and further supports the proposed electron transfer mechanism.

## 3 Conclusion

In summary, we have successfully constructed plasmonic Bi loaded BiVO_4_ via a simple in situ reduction method. The in situ formation of the plasmonic Bi on the BiVO_4_ can not only endow a intimate contact interface between the Bi and BiVO_4_, but also create the Bi─O─V covalent bridge, which can significantly facilitate the migration of the photogenerated hot electrons from Bi to BiVO_4_. Therefore, the optimized Bi/BiVO_4_ demonstrates an excellent photogenerated charge carrier separation efficiency, allowing more photogenerated charge carrier to participate in surface water oxidation reaction. As a consequence, the Bi/BiVO_4_‐450 demonstrates a high photocatalytic water oxidation performance, reaching an oxygen production rate of 4567.94 µmol h^−1^ g^−1^ under visible‐light irradiation. More importantly, it also shows a capability to be photoexcited by the low‐energy near‐infrared light irradiation, implying its potential to utilize the whole spectrum of incident solar energy. This work is anticipated to push forward the practical applications of the photocatalytic water oxidation in various commercial scenarios.

## 4 Experimental Section

### Synthesis of Bi/BiVO_4_

Polyhedral BiVO_4_ was synthesized via a one‐step hydrothermal technique. In a typical synthesis procedure, Bi(NO_3_)_3_•5H_2_O (5 M) was added into of HNO_3_ (2 m, 30 mL) under stirring for 30 min to obtain solution 1. Solution 2 was prepared by adding NH_4_VO_3_ (5 m) into NaOH (2 m, 30 mL) under stirring for 30 min. Subsequently, solution 2 was added to solution 1 and kept stirring for 2 h. The mixture was then transferred to stainless steel autoclave and heated at 180 °C for 24 h. After cooling, the product was centrifuged, washed three times, and dried to obtain the BiVO_4_. To prepare Bi/BiVO_4_, the obtained BiVO_4_ was calcined in N_2_/H_2_ atmosphere at different temperature of 400, 450, and 500 °C, and the resultant samples were labeled as Bi/BiVO_4_‐400, Bi/BiVO_4_‐450, and Bi/BiVO_4_‐500.

## Conflict of Interest

The authors declare no conflict of interest.

## Author Contributions

L.L. and Z.C. contributed equally to this study, designed and completed the experiments, and wrote the paper. D.F., J.L., and J.Y. revised the manuscript, and analyzed the experimental results.

[Correction added on 16 June 2025, after first online publication: (Author Contributions has been changed from “L.L. and Z.C. conceived and initiated this study, designed and completed the experiments, and wrote the paper. D.F., J.L., and J.Y. revised the manuscript, and analyzed the experimental results.” to “L.L. and Z.C. contributed equally to this study, designed and completed the experiments, and wrote the paper. D.F., J.L., and J.Y. revised the manuscript, and analyzed the experimental results.”]

## Supporting information



Supporting Information

## Data Availability

The data that support the findings of this study are available in the sup‐plementary material of this article.
