# Supplementary material for: Covalent Bridges in Bi Loaded BiVO4 Enabling Rapid Charge Transfer for Efficient Photocatalytic Water Oxidation
Source: Adv Sci (Weinh). 2025 Jun 5;12(31):e00666. doi: 10.1002/advs.202500666 (PMC12376518; doi:10.1002/advs.202500666)
Supplement: Supplementary file 1 — Supporting Information [file ADVS-12-e00666-s001.docx]

Supporting information

Covalent bridges in Bi loaded BiVO_4_ enabling rapid charge transfer for efficient photocatalytic water oxidation

Liyang Li^1#^, Zhiming Chen^2#^, Dong Fang^1*^, Jingxiang Low^2*^, Jianhong Yi^1*^

^1^Faculty of Materials Science and Engineering, Kunming University of Science and Technology, Kunming 650093, P.R. China.

^2^School of Physical Science and Technology, Tiangong University, Tianjin 300387, P.R. China.

**Note S1. Materials Characterizations**

X-ray powder diffraction (XRD) analysis of all photocatalysts was performed using a BDX320 instrument, scanning in the range of 10^°^ to 80^°^ at a rate of 10^°^min^-1^. Rietveld refinement was carried out using GSAS-II software. Surface and internal structural information of the materials were obtained through scanning electron microscopy (SEM) (JSM-7500F, JEOL, Japan) equipped with energy dispersive X-ray spectroscopy (EDS) and transmission electron microscopy (TEM) (Titan G2 60-300, FEI, United States of America). On an aberration-corrected TEM operated at 300 kV, high-angle annular dark-field scanning transmission electron microscopy (HAADF-STEM) images and elemental electron energy loss spectroscopy (EELS) maps were acquired. X-ray photoelectron spectroscopy (XPS) was conducted to study the chemical states of the samples using a PHI-5000C ESCA system with a Mg/Kα source. The obtained XAFS data was processed in Athena for background, pre-edge line and post-edge line calibrations. Then Fourier transformed fitting was carried out in Artemis Synchrotron Rad. The k3 weighting, k-range of 3-10.5 Å^-1^ and R range of 1-2 Å were used for the fitting of Bi_2_O_3_; The k3 weighting, k-range of 3-10.5 Å^-1^ and R range of 1-4 Å were used for the fitting of Bi/BiVO_4_-450. Additionally, optical properties were measured using ultraviolet-visible diffuse reflectance spectroscopy (DRS) (Shimadzu, UV-3600, Japan) and photoluminescence (PL) spectra at 371 nm were obtained using a Shimadzu RF-5301PC spectrophotometer. Time-resolved photoluminescence (TRPL) measurements were carried out at 371 nm using an Edinburgh FLS1000 spectrometer (Edinburgh, Instruments, United Kingdom of Great Britain and Northern Ireland). The surface area of the samples was determined using nitrogen adsorption and the Brunauer-Emmet-Teller (BET) method with the US ASAP (Accelerated Surface Area and Porosimetry) 2020 system. Using a He Iα (hυ = 21.2 eV) radiation source, measurements of UV photoelectron spectroscopy (UPS) (ThermoFischer, ESCALAB 250Xi, United States of America) were carried out. At room temperature, a hemispherical electron energy analyzer was utilized to conduct the angle-resolved UPS (ARUPS). Electron spin resonance (ESR) was measured on a Bruker A300 spectrometer (Karlsruhe, Germany). The time-resolved transient absorption spectra were based on a regenerative amplified Ti: sapphire laser system from Coherent (800 nm, 35 fs, 6 mJ/pulse, and 1 kHz repetition rate), nonlinear frequency mixing techniques and the Helios spectrometer (Ultrafast Systems LLC). The three-dimensional finite-difference time-domain (FDTD) method was used to simulate the near-field electric field distribution of Bi/BiVO_4_-450. In this simulation, Bi nanoparticles were evenly dispersed on the x-y plane of the cross-section of BiVO_4_ and embedded in water.

**Note S2. DFT Calculations**

Density functional theory calculations were performed using the Vienna Ab initio Simulation Package (VASP) and the Projector Augmented-Wave (PAW) method. The exchange-correlation potential was represented by the Perdew-Burke-Ernzerhof (PBE) functional within the Generalized Gradient Approximation (GGA). A heterostructure consisting of 147 atoms in the Bi(002)/BiVO_4_(001) interface was constructed. A vacuum layer of 15 Å thickness was included along the z-direction. The cutoff energy was set to 500 eV. The k-point sampling grids for the Bi(002)/BiVO_4_(001) heterostructure, bulk BiVO_4_, and bulk Bi were set to 2×4×2, 5×2×5, and 1×2×1, respectively. An effective U value of 4.5 eV was selected for the 3d orbitals of vanadium atoms. The structures were relaxed until the convergence criteria for energy and forces were below 1.0×10^−5^ eV/atom and 4.0×10^−2^ eV/Å, respectively. A vacuum layer of 15 Å was used. The van der Waals interactions were described using the DFT-D3 method.

**Note S3. Photoelectrochemical and electrochemical tests**

Electrochemical photocurrent response (i-t), electrochemical impedance spectroscopy (EIS), linear sweep voltammetry (LSV), and Mott-Schottky (M-S) tests were conducted on an electrochemical analyzer equipped with a standard three-electrode system. A 0.5 M Na_2_SO_4_ aqueous solution was used as the electrolyte in this system. Platinum foil, an Ag/AgCl electrode, and ITO glass with a coated area of 2.0×2 cm^2^ were chosen as the electrode, reference electrode, and working electrode, respectively. Visible light was simulated using a 300 W xenon lamp.

**Note S4. Preparation of working electrode**

The ITO substrate is ultrasonically cleaned with deionized water and absolute ethanol. Next, in a 5 mL glass container, 450 μL of ethanol is thoroughly mixed with 50 μL of Nafion solution, followed by the addition of 10 mg of photocatalytic powder, which is stirred uniformly. After low-power ultrasonic dispersion for 15 min, the mixture is continuously stirred for 24 h to ensure a homogeneous solution. Finally, 20 μL of the mixed solution is evenly coated onto the effective working area (2×2 cm^2^) of the ITO substrate using a pipette and is dried at 60 °C for 12 h to complete the preparation of the working electrode.

**Note S5. Photocatalytic measurement**

Photocatalytic O_2_ production process takes place in a glass gas-closed circulation system (CEL-PAEM, D8PRO, China). In each photocatalytic experiment, 50 mg of catalyst is stirred and dispersed in 80 mL of 0.05 M AgNO_3_ aqueous solution, while the temperature of the reaction solution is controlled at 6 °C using cooling water. Prior to light irradiation, the reaction system is evacuated multiple times to remove internal air, bringing the reactor pressure to 0.08 MPa. A combination of a 300 W xenon lamp and a 420 nm filter (CELHXF300-T3) provides visible light irradiation. The amount of oxygen produced is measured using an online gas chromatograph (GC9790Ⅱ, with a TCD detector and nitrogen carrier gas). The AQY is calculated from the ratio of the number of electrons reacted to the number of incident photons during the water splitting process. The calculation formula is:

*AQY=*$\frac{\text{4}\text{×n(O}\text{2}\text{)×N}\text{A}\text{×h×c}}{\text{F×S×T×}\text{λ}}\times$100%

where *n*(*O_2_*) refers to the hydrogen evolution (mol), *T* is the iradiaion time (s), *N_A_* is the Avogadro constant (6.022×10^23^ mol^-1^). Erefers to the total energy of the incident photon (J), *F* refers to the average spectral irradiance (w/cm^2^), *S* is the irradiation area (19.62 cm^2^ in this paper), *λ* is the wavelength of monochromatic light (m), *h* is the Planck constant (6.626×10^-34^ J·s) and *c* is the ight speed (3.0×10^8^ m s^-1^).

**Note S6.** **Synthesis of Bi/BiVO_4_-CR**

450 μL of HNO_3_ was added into 100 ml of deionized water. After stirring evenly, 2.7 g of Bi(NO_3_)_3_·5H_2_O had been added and completely dissolved. While stirring, 50 mg of the hydrothermally prepared BiVO_4_ powder had been added, followed by 50 mg of sodium borohydride (NaBH_4_). After the reaction had been completed, the solution had been centrifuged, washed and dried. The product Bi/BiVO_4_-CR had been obtained by calcining at 450 ℃.

**Note S7. Chemicals and materials**

All reagents for the preparation of BiVO_4_ and Bi/BiVO_4_, such as ammonium metavanadate (NH_4_VO_3_), sodium hydroxide (NaOH), nitric acid (HNO_3_), sodium borohydride (NaBH_4_) and bismuth nitrate pentahydrate (Bi(NO_3_)_3_·5H_2_O), were purchased from Shanghai McLean Biochemical Technology Co. Ltd and used as received without further purification.

**Figure S1**. (a) XRD and (b) magnified XRD patterns of Bi/BiVO_4_-400, Bi/BiVO_4_-450 and Bi/BiVO_4_-500.


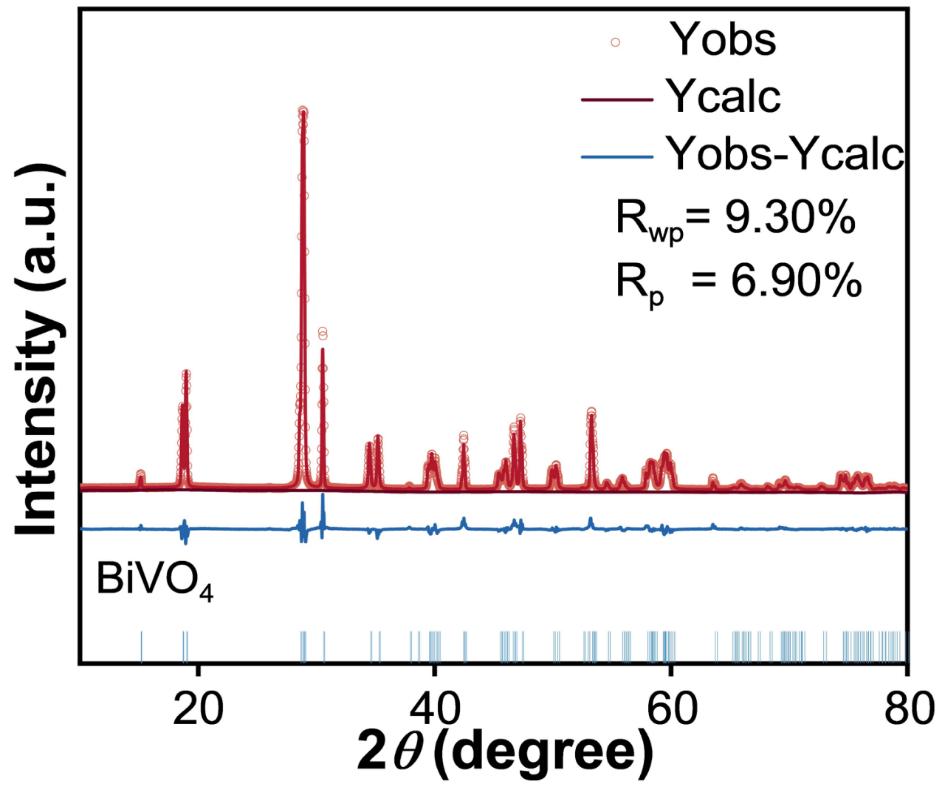


**Figure S2.** XRD refinement result of BiVO_4_.


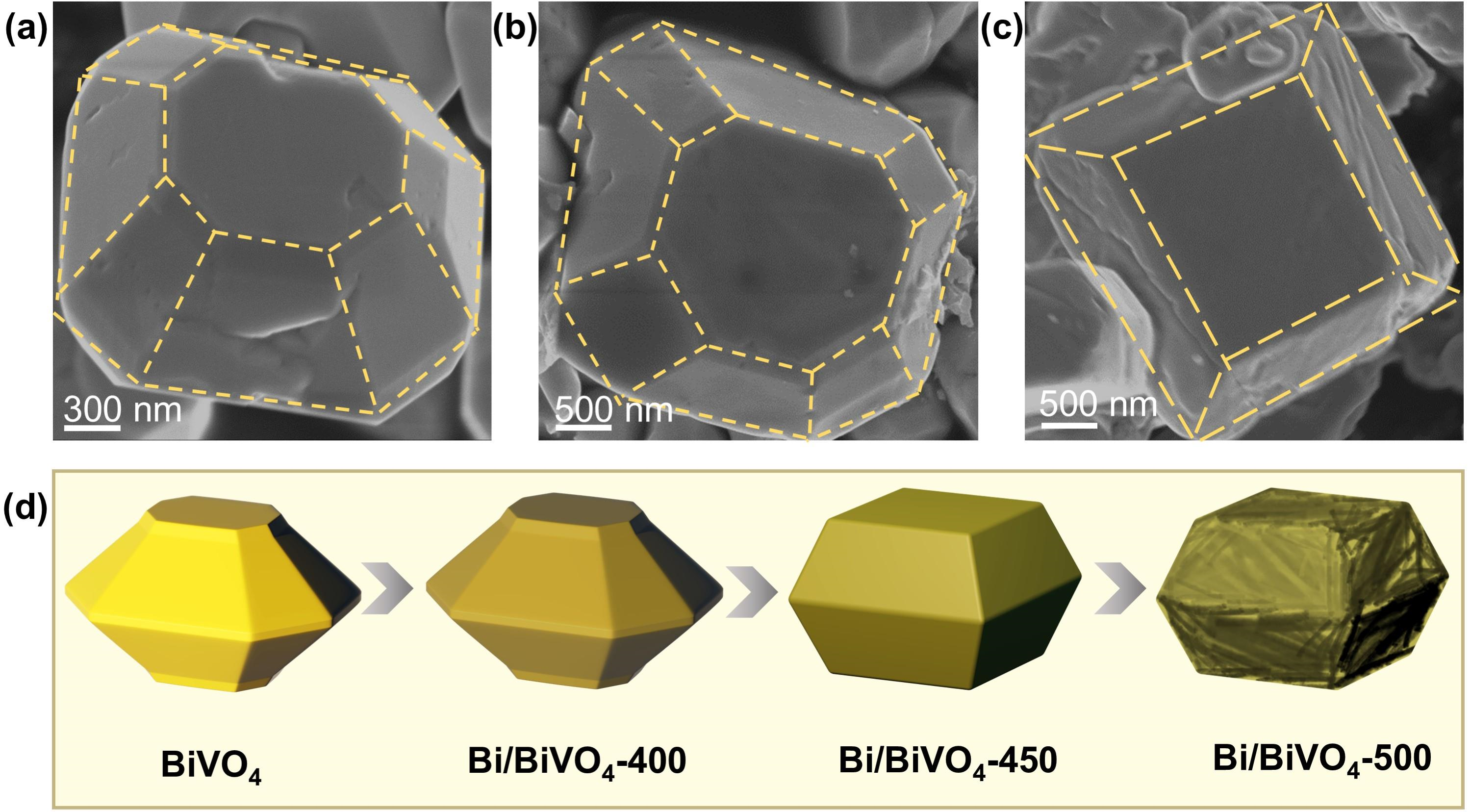


**Figure S3**. (a–c) SEM images of BiVO_4_ (a), Bi/BiVO_4_-400 (b) and Bi/BiVO_4_-500 (c). (d) Schematic illustration of the morphological evolution of BiVO_4_, Bi/BiVO_4_-400, Bi/BiVO_4_-450 and Bi/BiVO_4_-500.

**Figure S4**. SEM image of Bi/BiVO_4_-450.


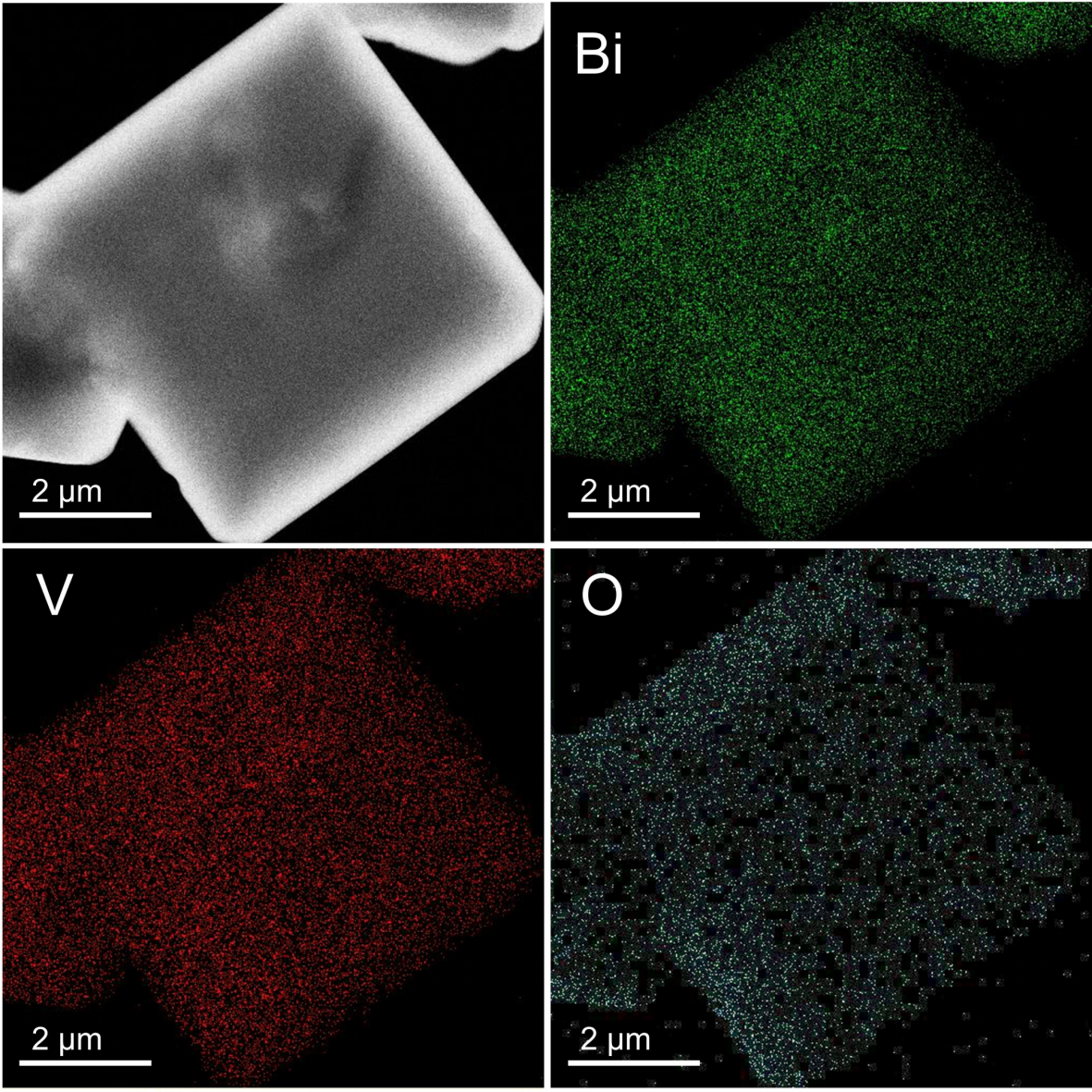


**Figure S5.** EDS mapping images for Bi, V and O elements on Bi/BiVO_4_-450.


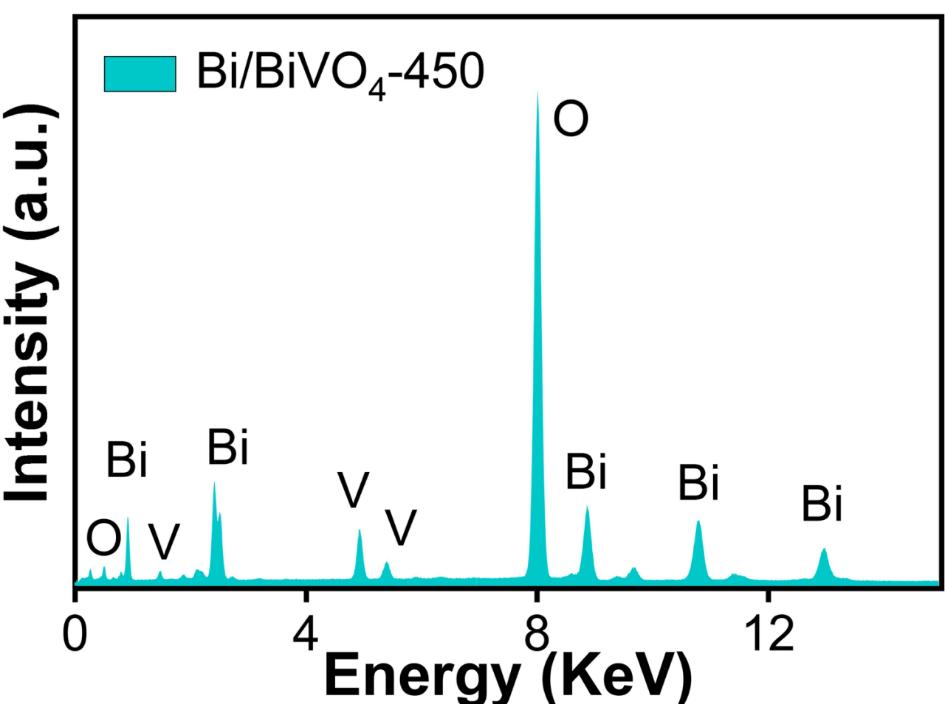


**Figure S6.** EELS spectrum of Bi/BiVO_4_-450.


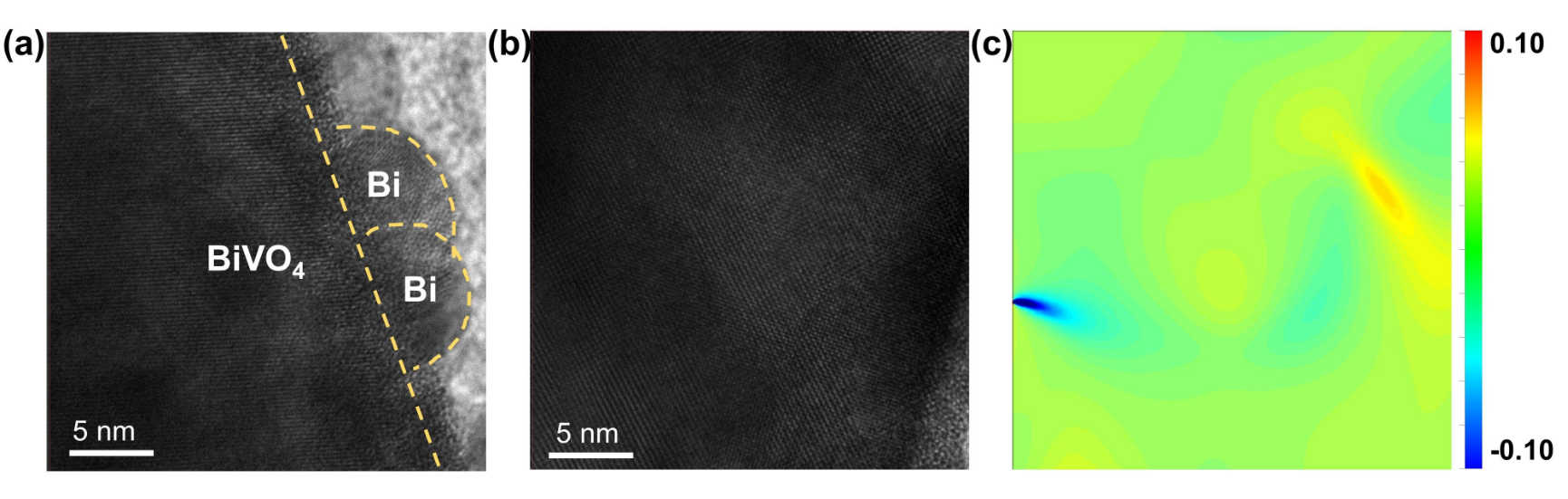


**Figure S7**. (a,b) TEM images of Bi/BiVO_4_-450 (a) and BiVO_4_ (b). (c) Corresponding GPA strain map image of BiVO_4_, showing ε_xy_ component.


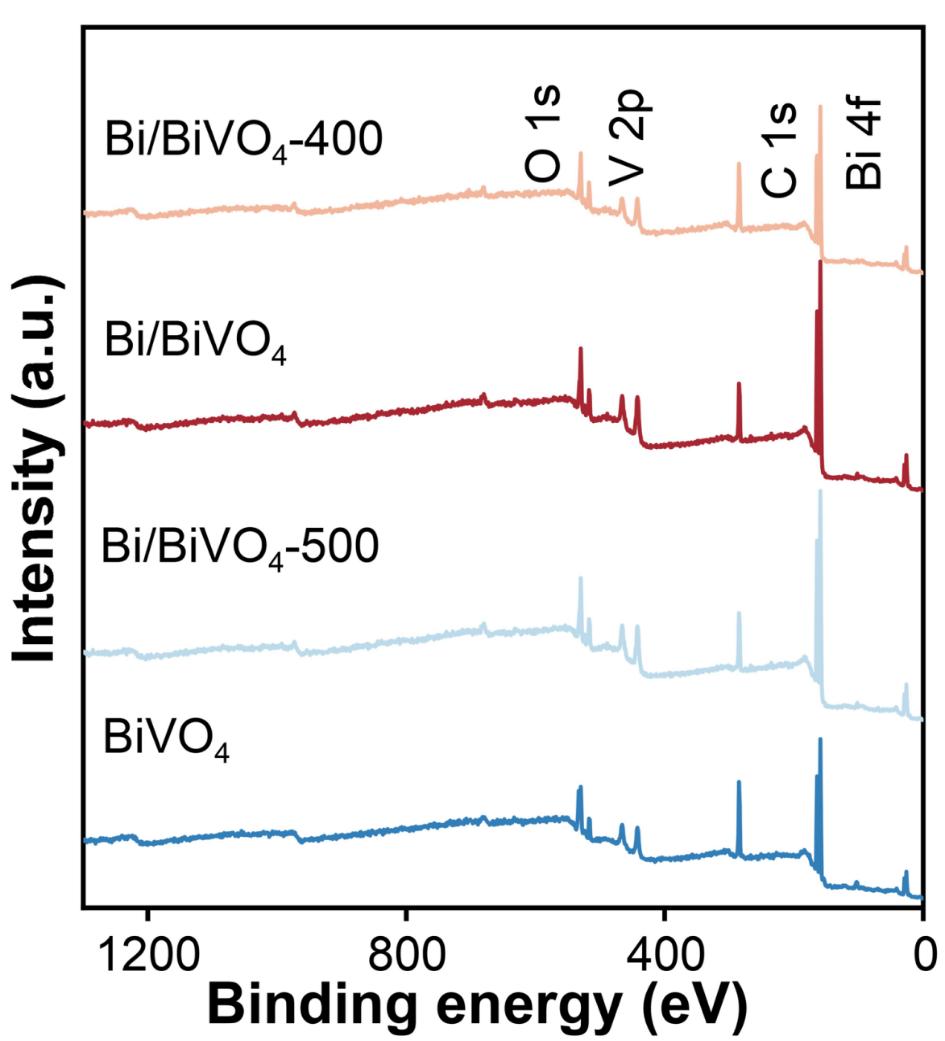


**Figure S8.** XPS survey spectra of BiVO_4_, Bi/BiVO_4_-400, Bi/BiVO_4_-450 and Bi/BiVO_4_-500.


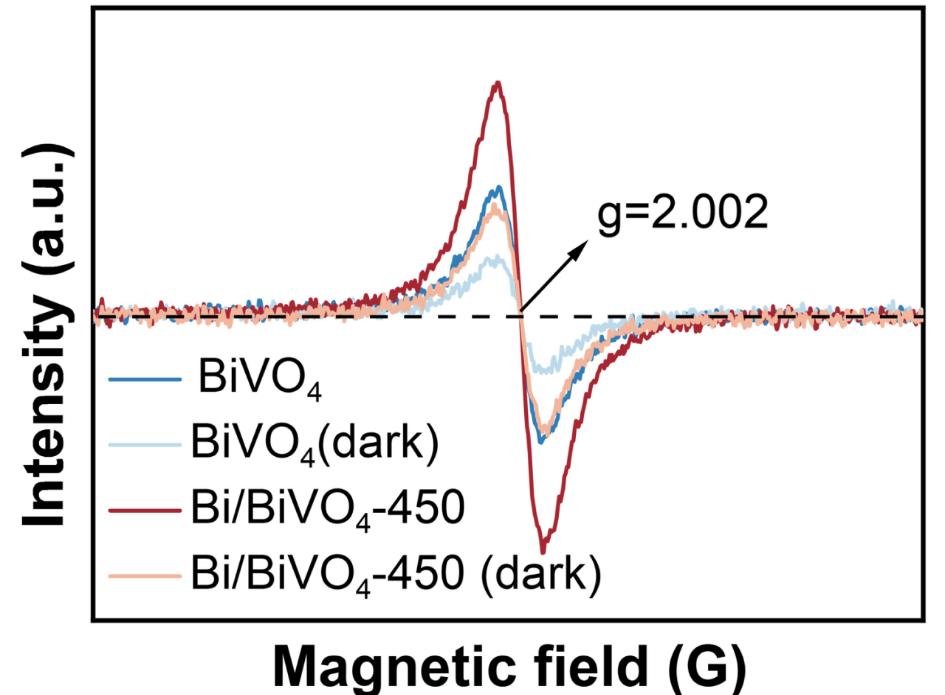


**Figure S9.** ESR spectra of BiVO_4_ and Bi/BiVO_4_-450.

**Figure S10.** (a) Bi K-edge radial distance K space spectra. (b) Fitting curves of Fourier transform (FT) extended X-ray absorption fine structure (EXAFS) at the Bi K-edge K space of Bi/BiVO_4_-450. (c,d) Fitting curves of Fourier transform (FT) extended X-ray absorption fine structure (EXAFS) at the Bi K-edge R space (c) and *k* space (d) of Bi_2_O_3_.

**Figure S11**. (a) N_2_ adsorption-analytical isotherm and (b) aperture size distribution plots of BiVO_4_ and Bi/BiVO_4_-450.


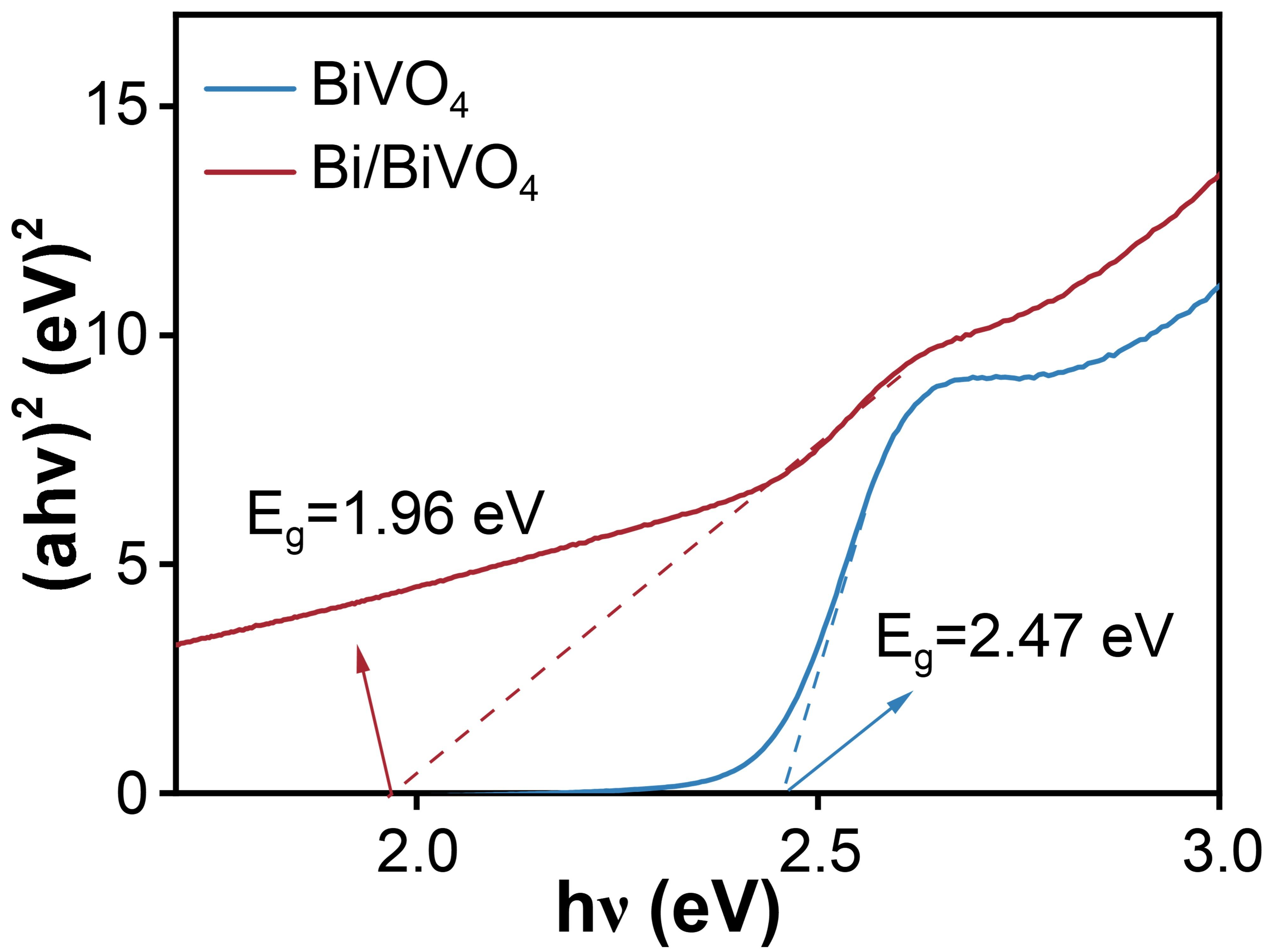


**Figure S12.** The bandgap calculation diagram of BiVO_4_ and Bi/BiVO_4_-450.


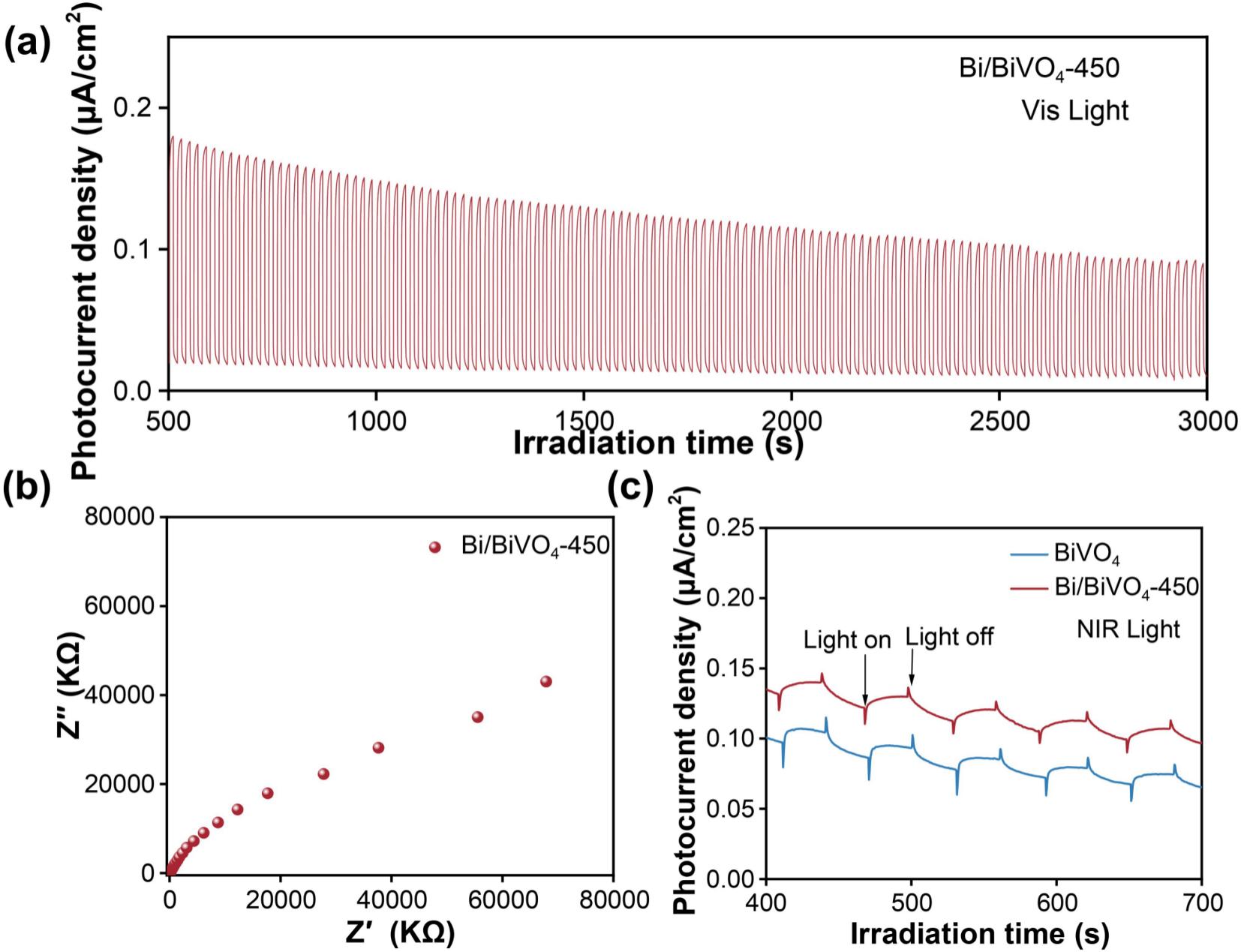


**Figure S13.** (a,b) Electrochemical transient photocurrent response of Bi/BiVO_4_-450 at 100 cycles (a) and EIS Nyquist plots of Bi/BiVO_4_-450 after 100 cycles of electrochemical transient photocurrent response (b). (c) Electrochemical transient photocurrent responses spectra of BiVO_4_ and Bi/BiVO_4_-450 under near-infrared light.


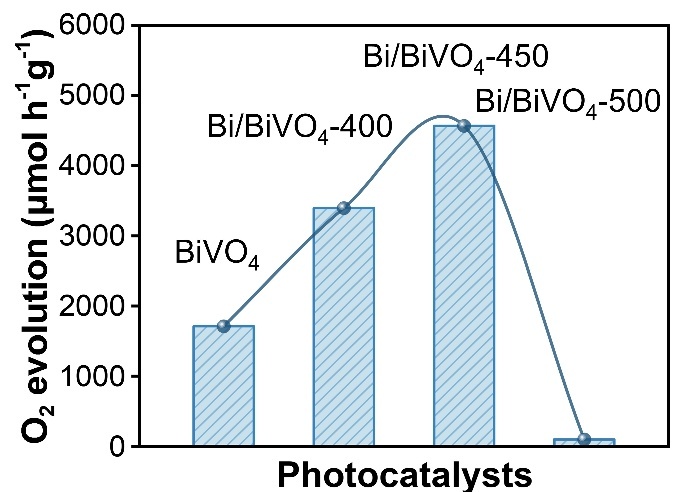


**Figure S14.** Photocatalytic O_2_ production production rate.


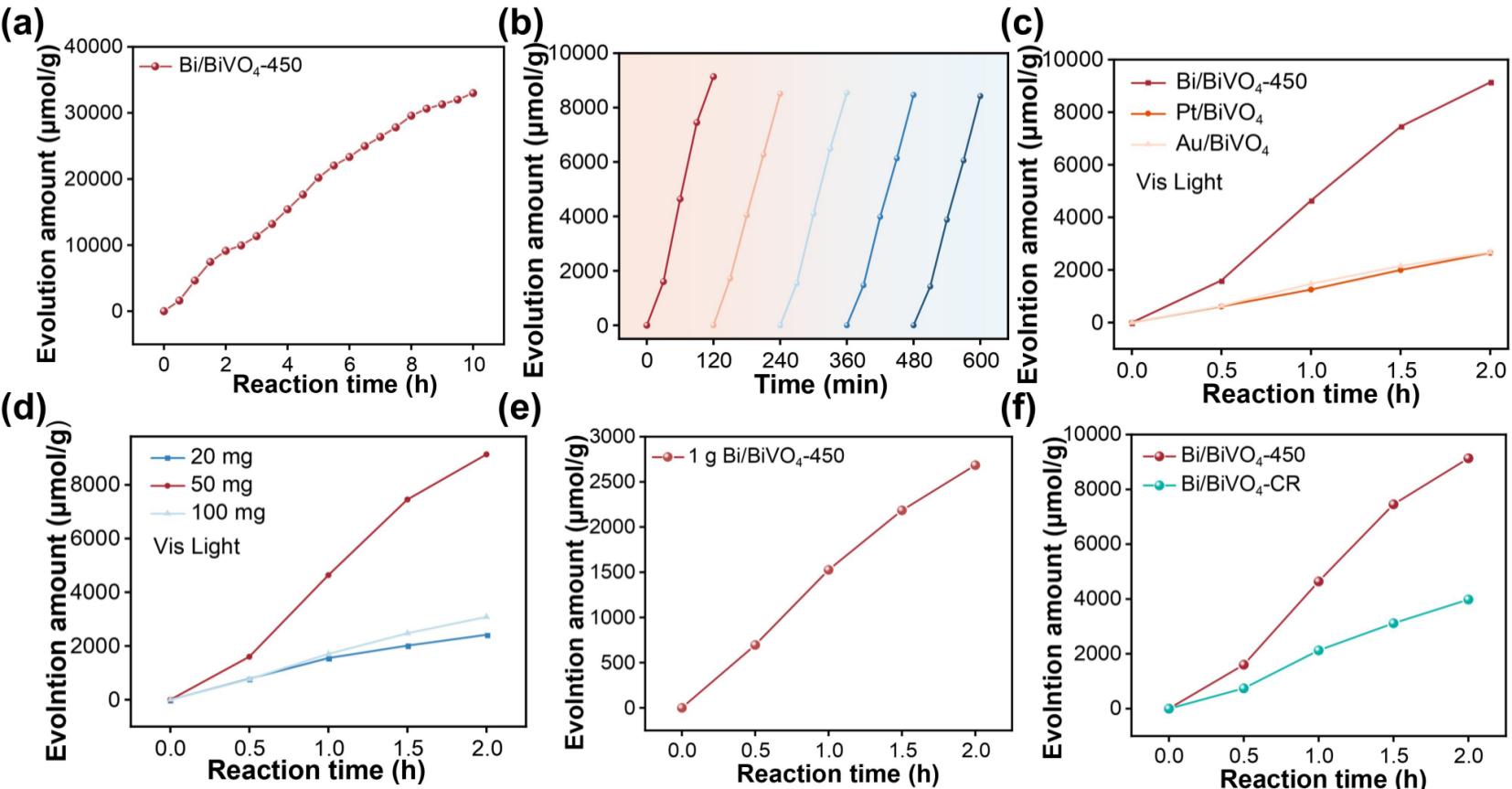


**Figure S15.** (a) Photocatalytic O_2_ production of Bi/BiVO_4_-450 under visible light (λ > 420 nm) irradiation for 10 h. (b) Photocatalytic O_2_ production during cyclic test over Bi/BiVO_4_-450. (c) Photocatalytic O_2_ production during cyclic test over Bi/BiVO_4_-450, Pt/BiVO_4_ and Au/BiVO_4_ under visible-light irradiation (λ > 420 nm). (d) Photocatalytic O_2_ production of 20 mg, 50 mg and 100 mg of Bi/BiVO_4_-450 under visible-light irradiation (λ > 420 nm). (e) Photocatalytic O_2_ production of 1 g of Bi/BiVO_4_-450 under visible-light irradiation (λ > 420 nm). (f) Photocatalytic oxygen production performance of Bi/BiVO_4_-450 and Bi/BiVO_4_-CR.


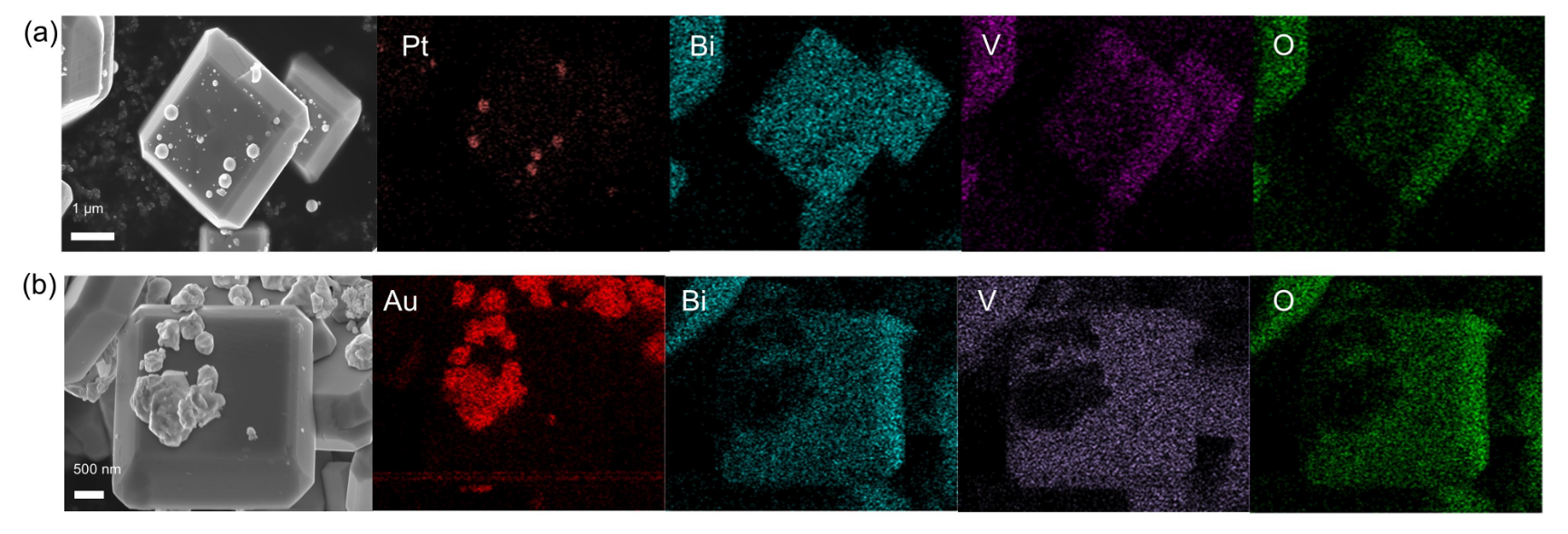


**Figure S16.** SEM and corresponding EDS images of (a) Pt/BiVO_4_ and (b) Au/BiVO_4_.


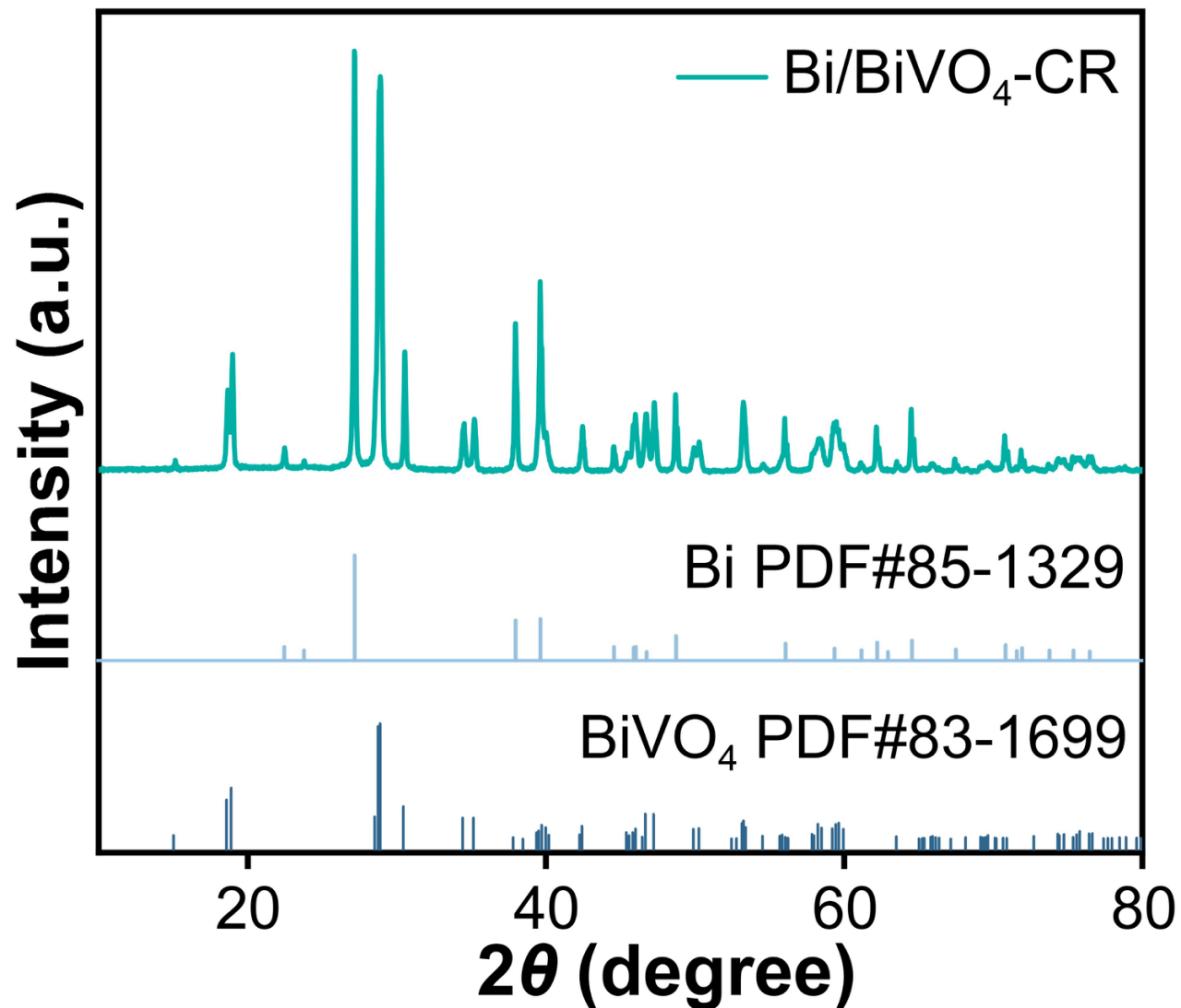


**Figure S17.** XRD pattern of Bi/BiVO_4_-CR.


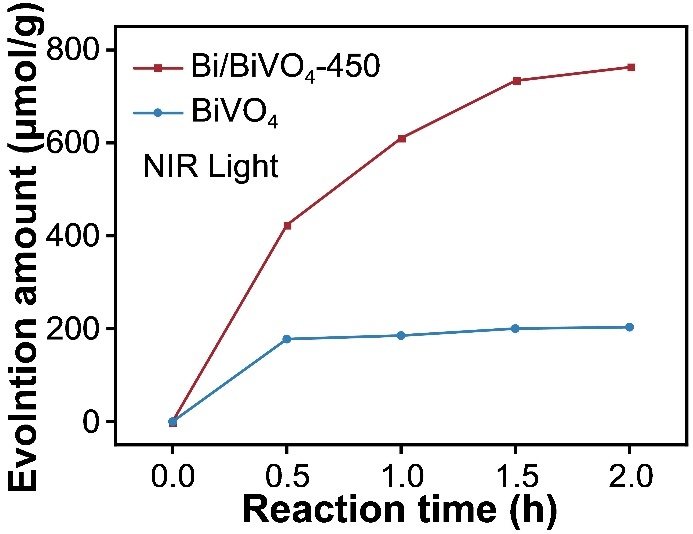


**Figure S18.** Photocatalytic O_2_ production of BiVO_4_ and Bi/BiVO_4_-450 under near-infrared light irradiation.


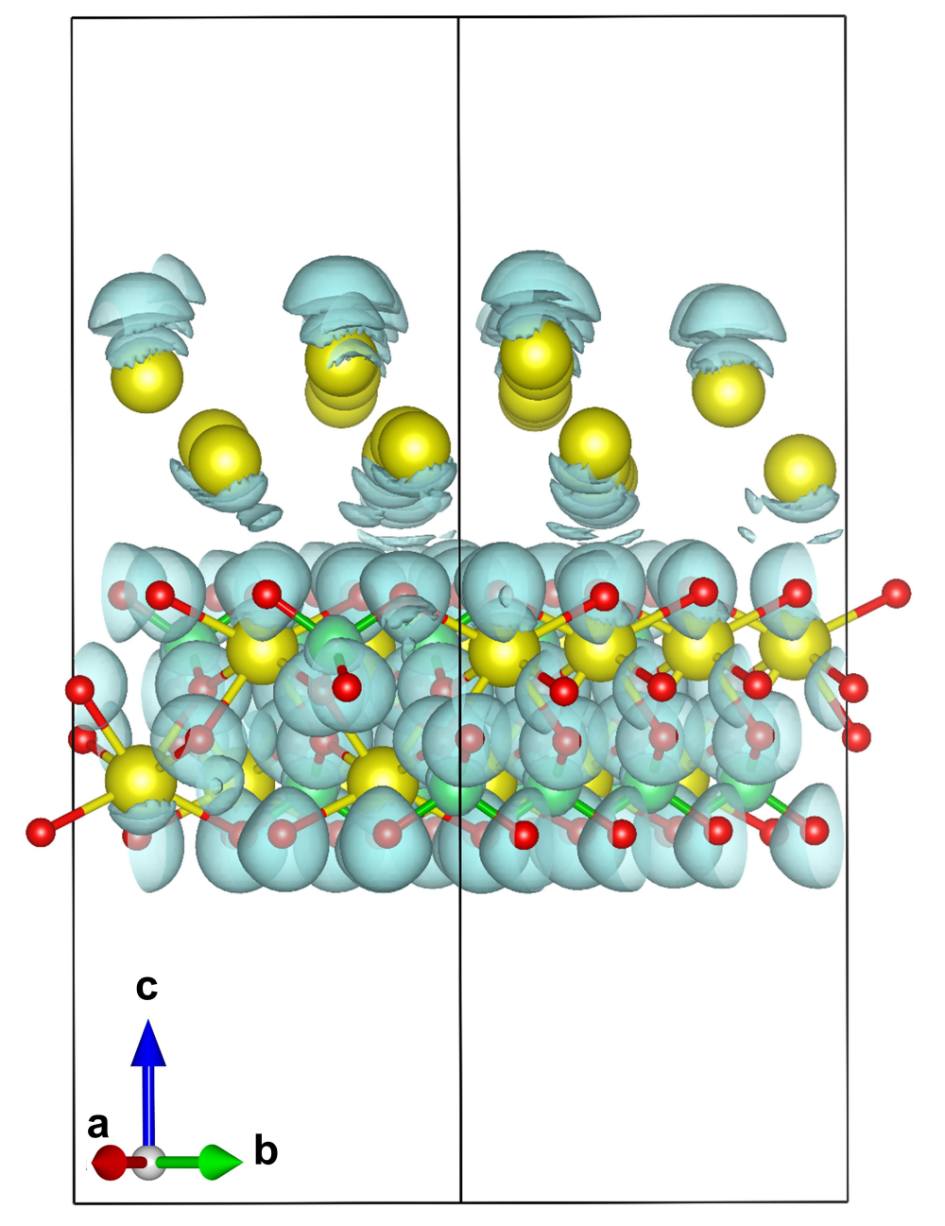


**Figure S19.** 3D structure diagrams of Bi/BiVO_4_ corresponding to the ELF calculation.


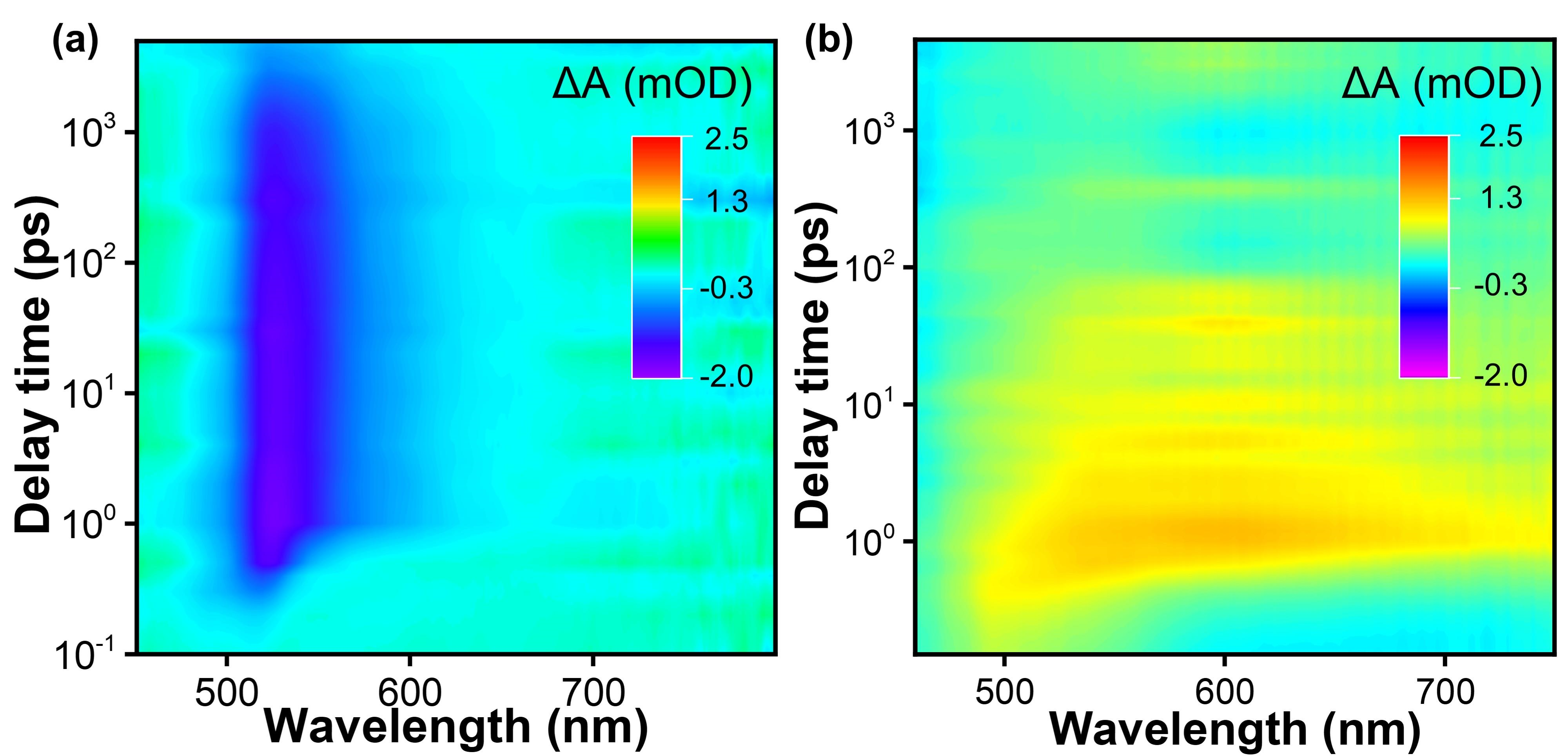


**Figure S20.** 2D pseudo-color plots of fs-TA spectra of (a) Bi and (b) BiVO_4_ under 380 nm fs laser pulses.


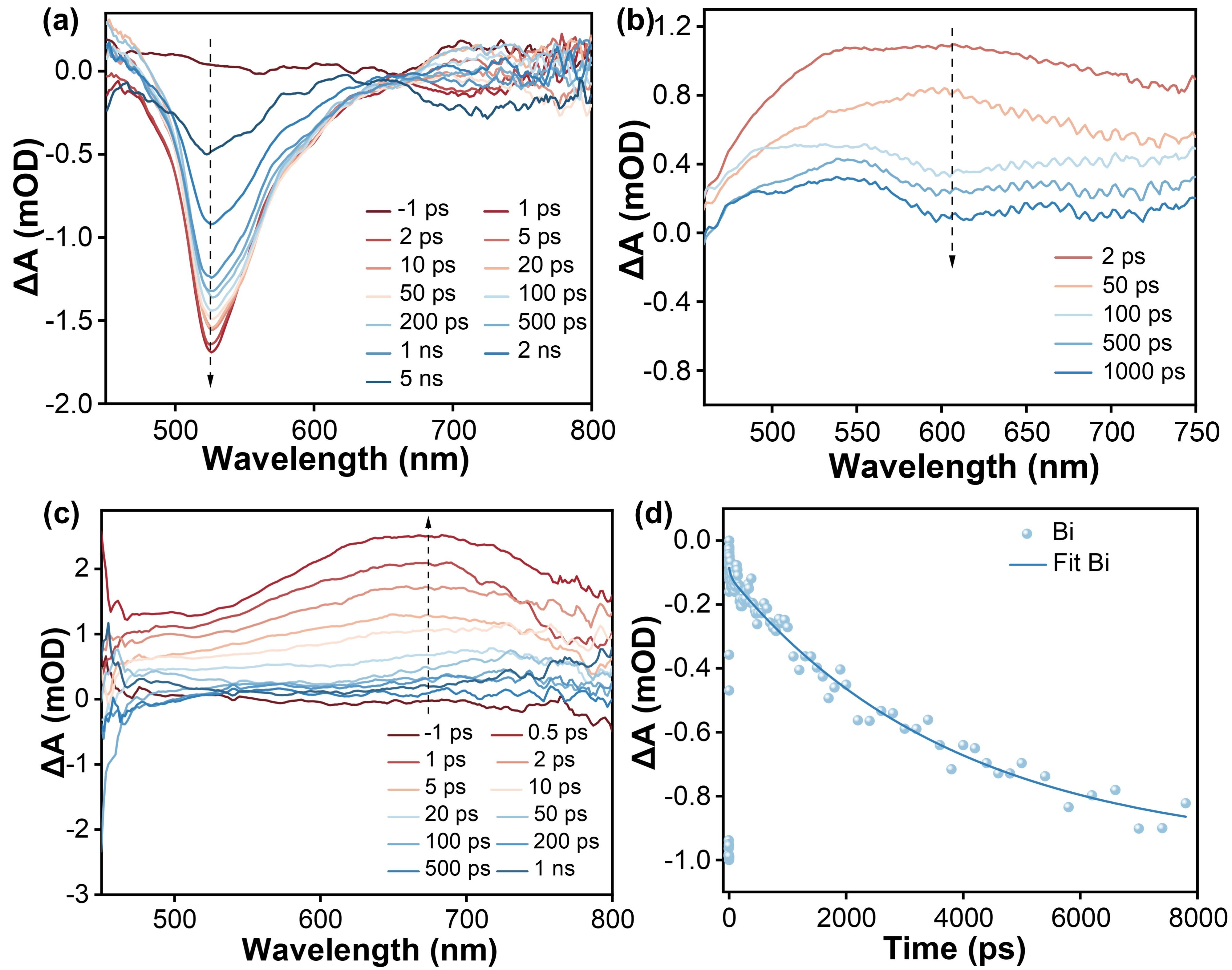


**Figure S21.** (a–c) Absorption decay from fs-TA spectra at different probe delays for Bi (a), BiVO_4_ (b) and Bi/BiVO_4_-450 (c). (d) Kinetics decay process from fs-TA spectra for Bi.


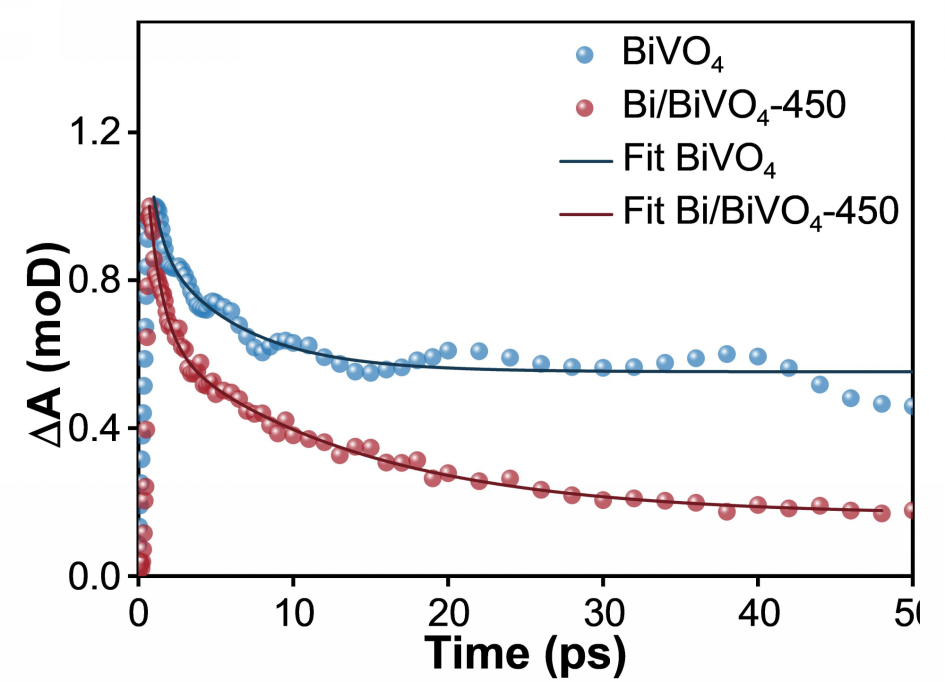


**Figure S22.** Kinetics decay process from fs-TA spectra for BiVO_4_ and Bi/BiVO_4_-450, and their corresponding dual-exponential decay fitted curves.


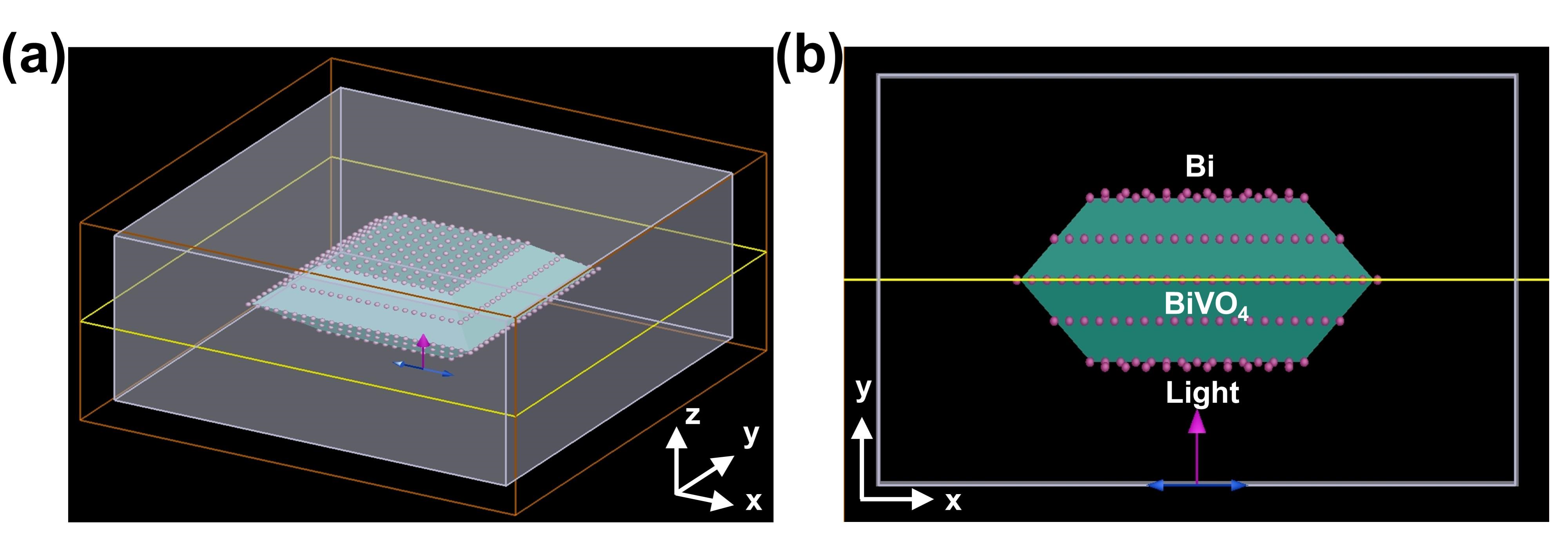


**Figure S23.** Finite-difference time-domain simulation model in (a) 3D view and (b) cross-sectional view in the X-Y plane for Bi/BiVO_4_-450.


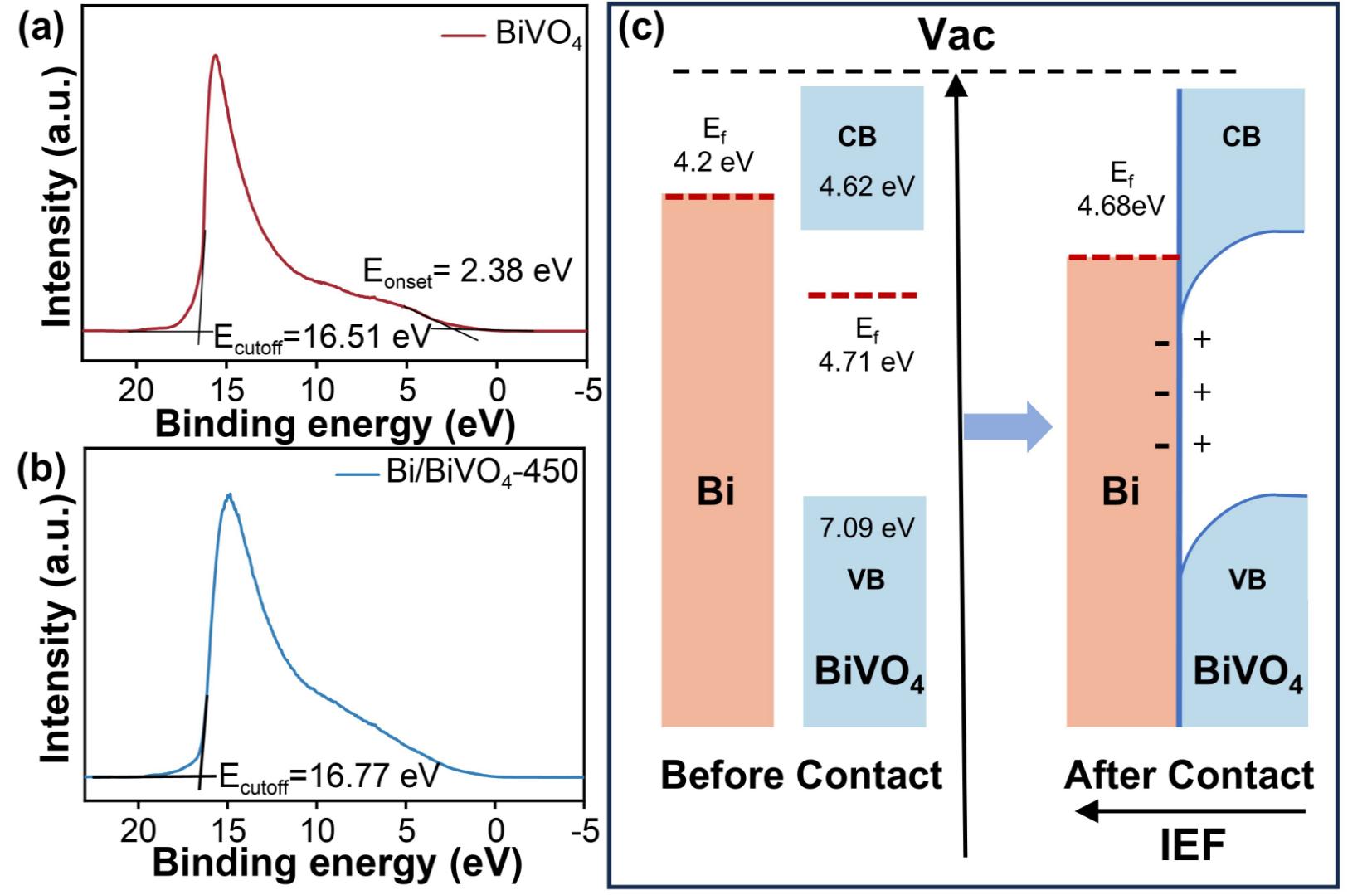


**Figure S24.** (a,b) UPS spectra of Bi/BiVO_4_-450 (a) and BiVO_4_ (b). (c) Schematic illustration of the interfacial Schottky barrier for the Bi/BiVO_4_-450.


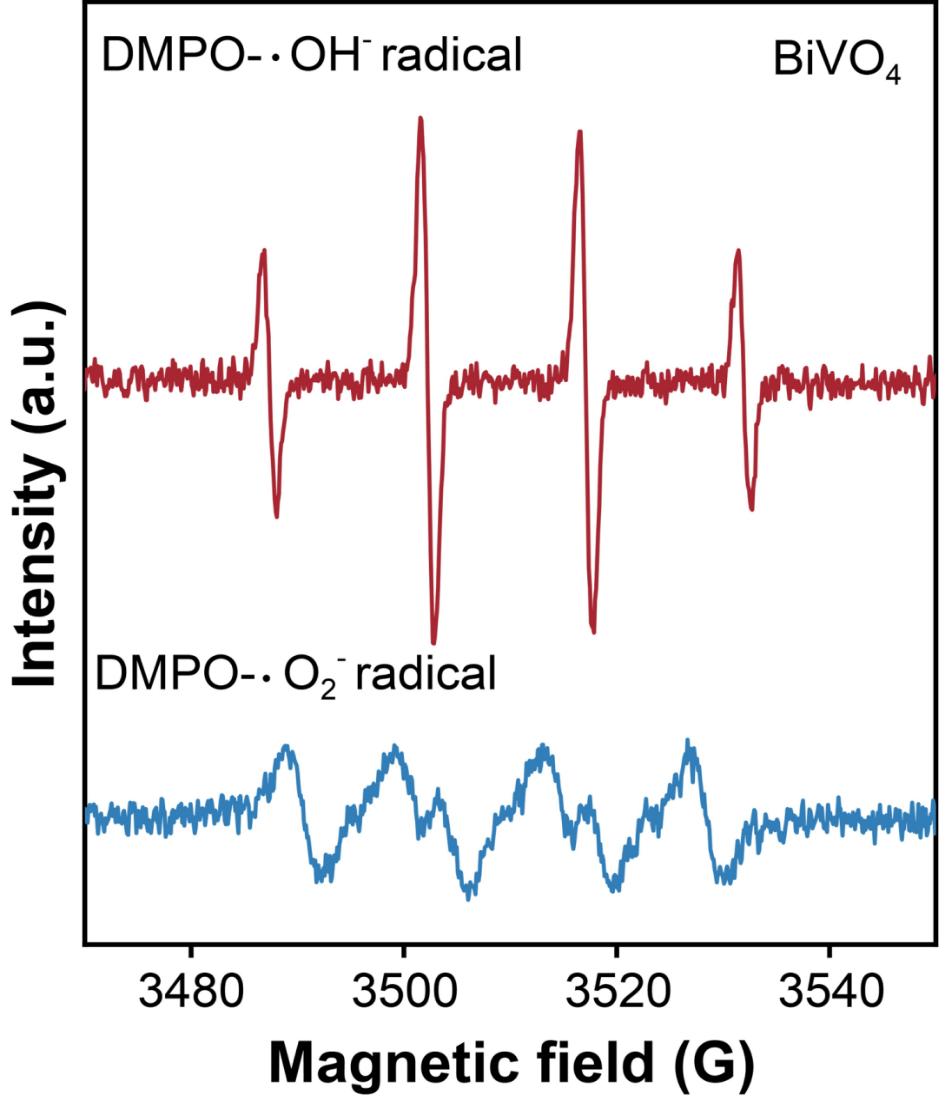


**Figure S25.** ESR signals of •O_2_^−^ radicals and •OH^−^ radicals of BiVO_4_ using DMPO as trapping agents.


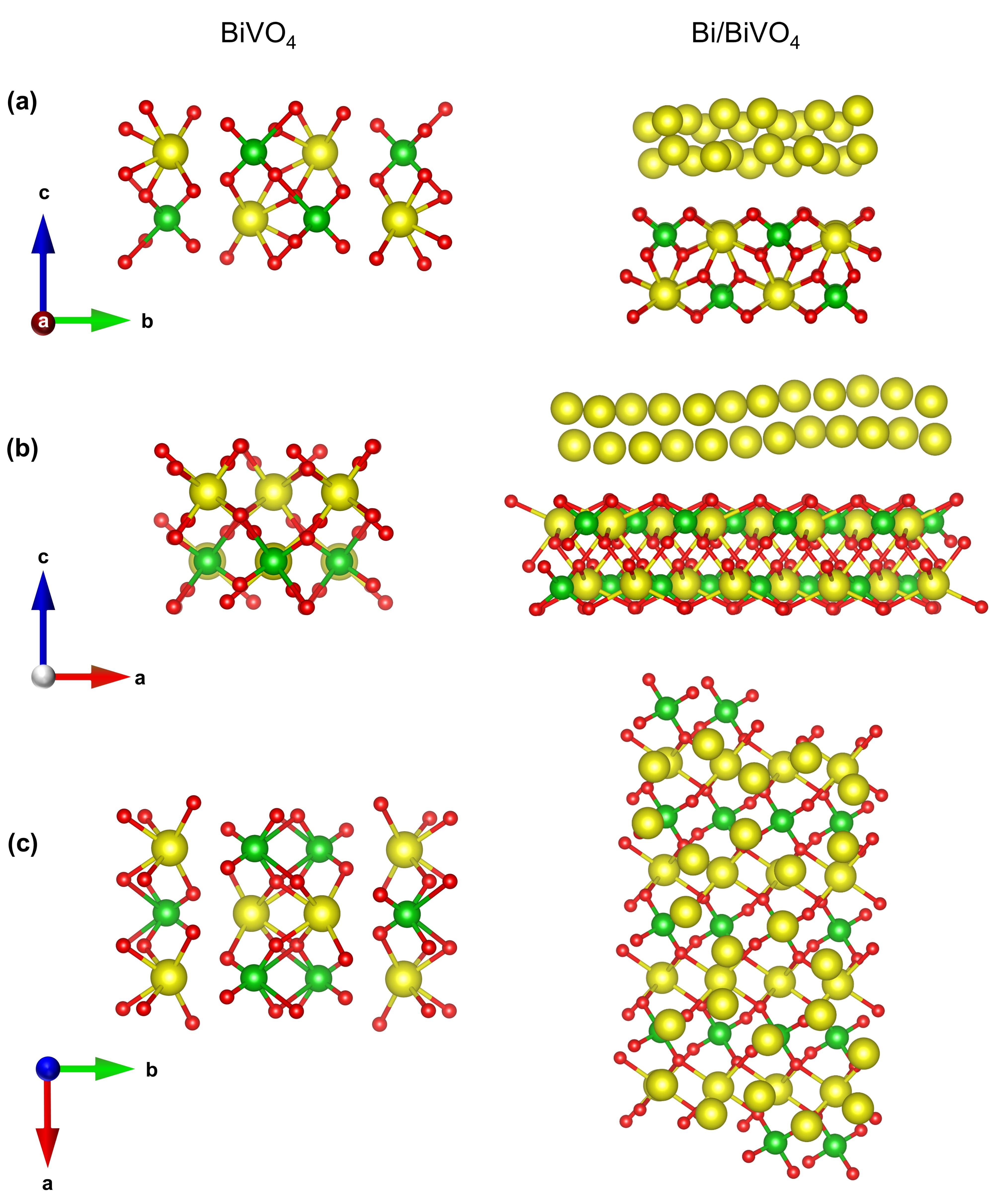


**Figure S26.** (a) Front, (b) side and (c) top views of BiVO_4_ and Bi/BiVO_4_.


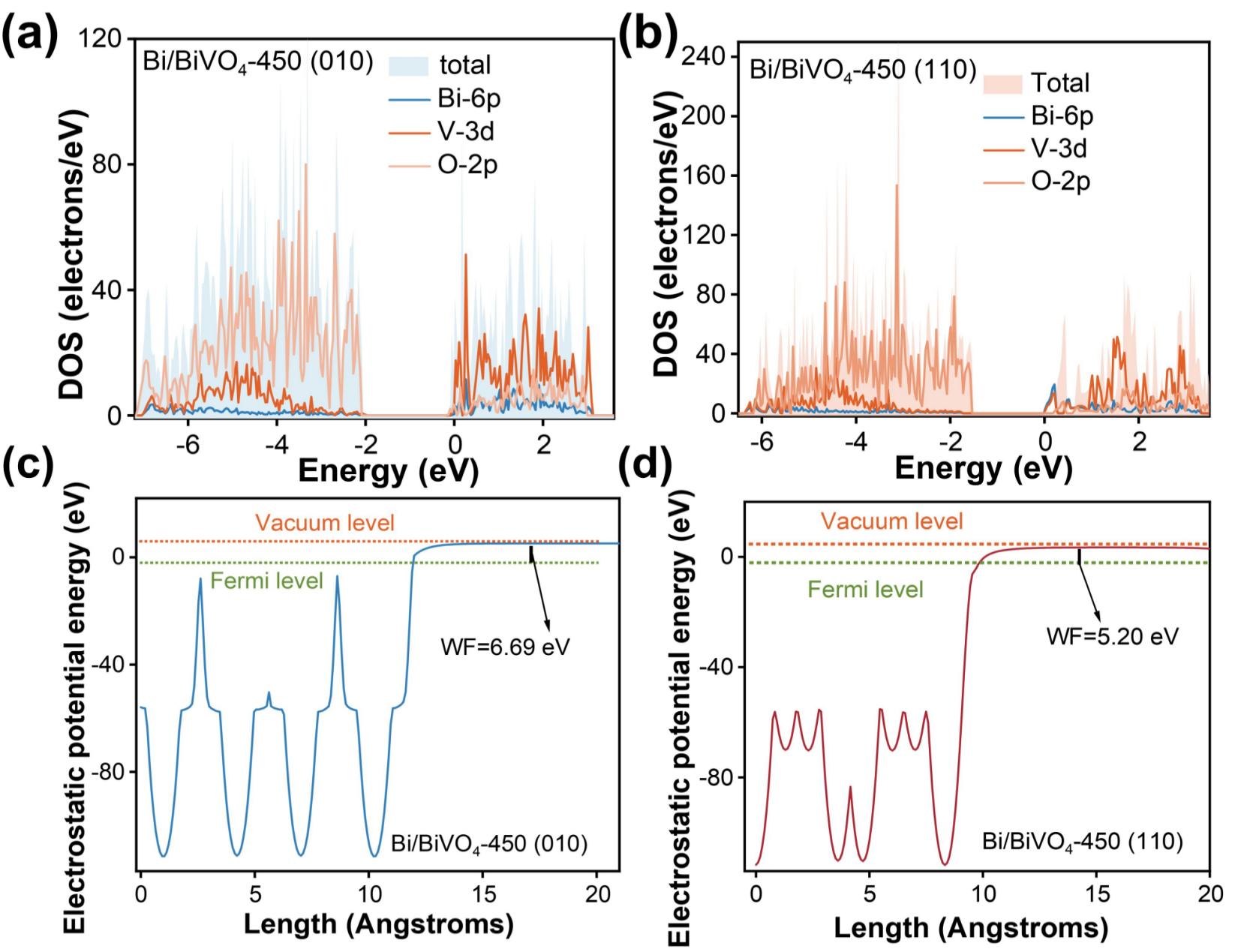


**Figure S27.** (a,b) Total and partial DOS of Bi/BiVO_4_-450 (010) (a) and Bi/BiVO_4_-450 (110) (b). (c,d) Work function calculation chart of Bi/BiVO_4_-450 (010) (c) and Bi/BiVO_4_-450 (110) (d).


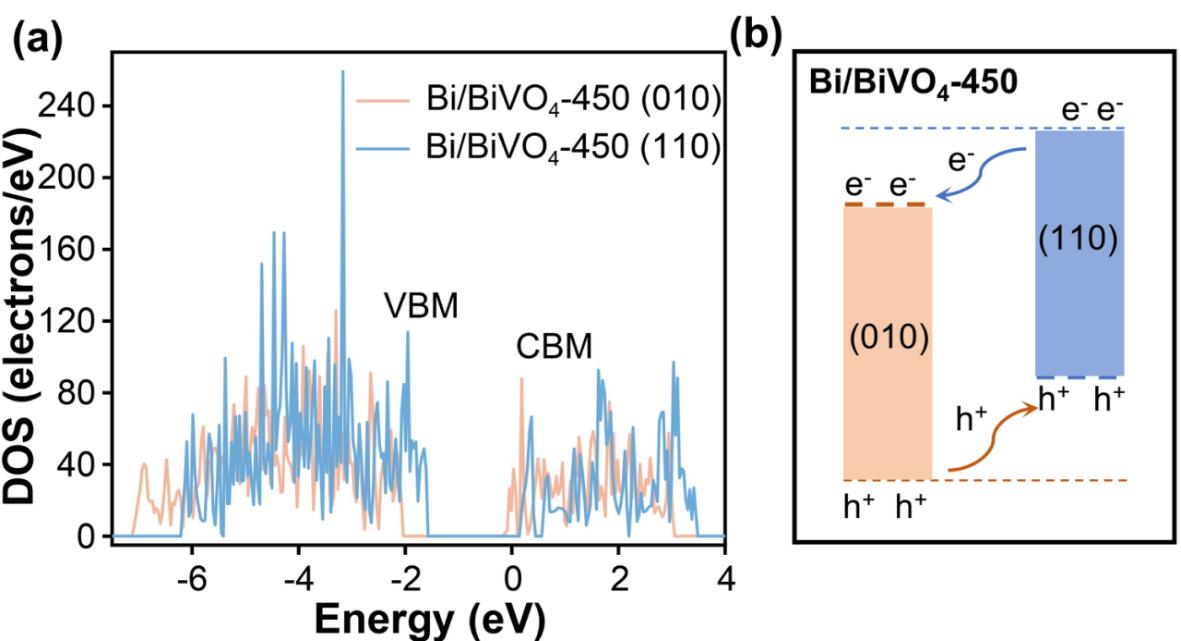


**Figure S28.** (a) Density of states and (b) schematic illustration of different energy levels for (010) and (110) planes of Bi/BiVO_4_-450.


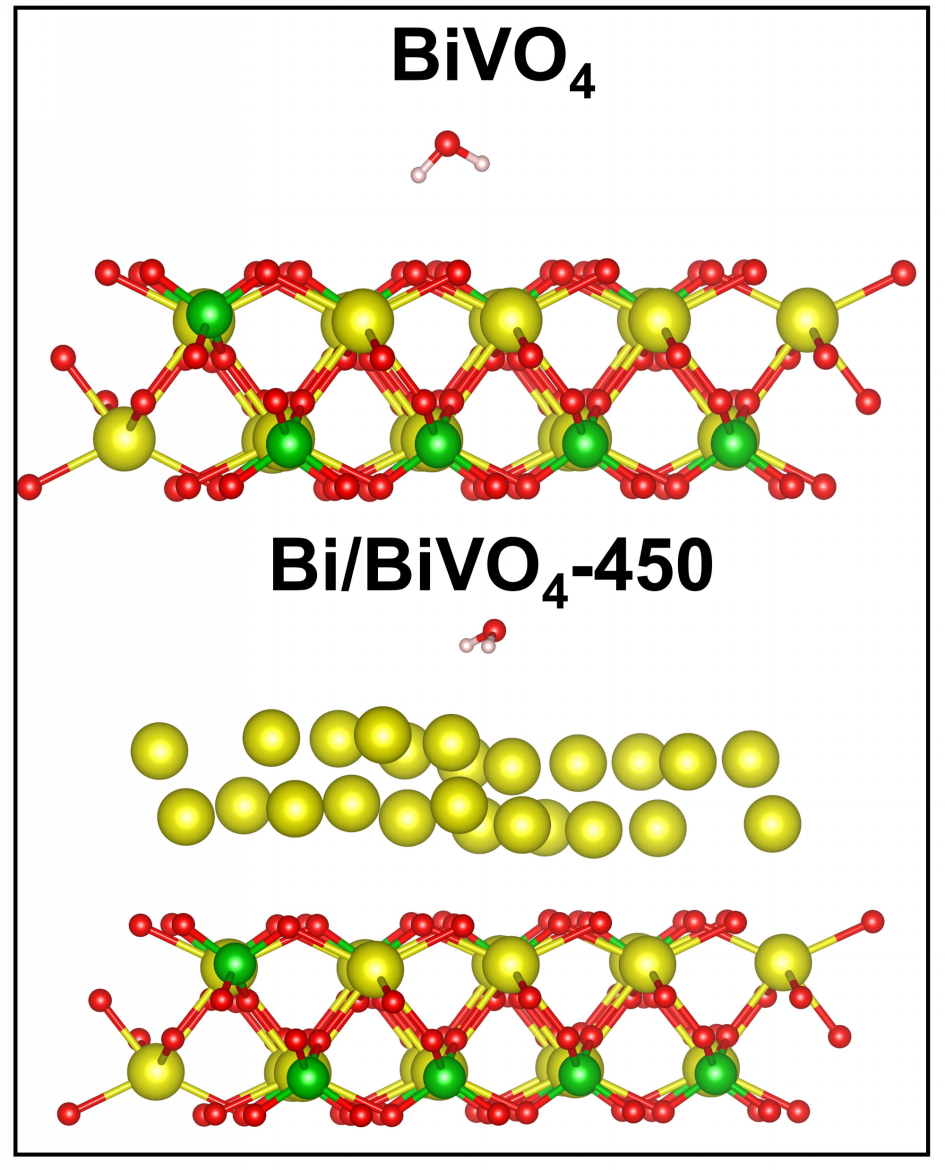


**Figure S29.** Optimal structure illustration of H_2_O molecule adsorbed on BiVO_4_ and Bi/BiVO_4_.


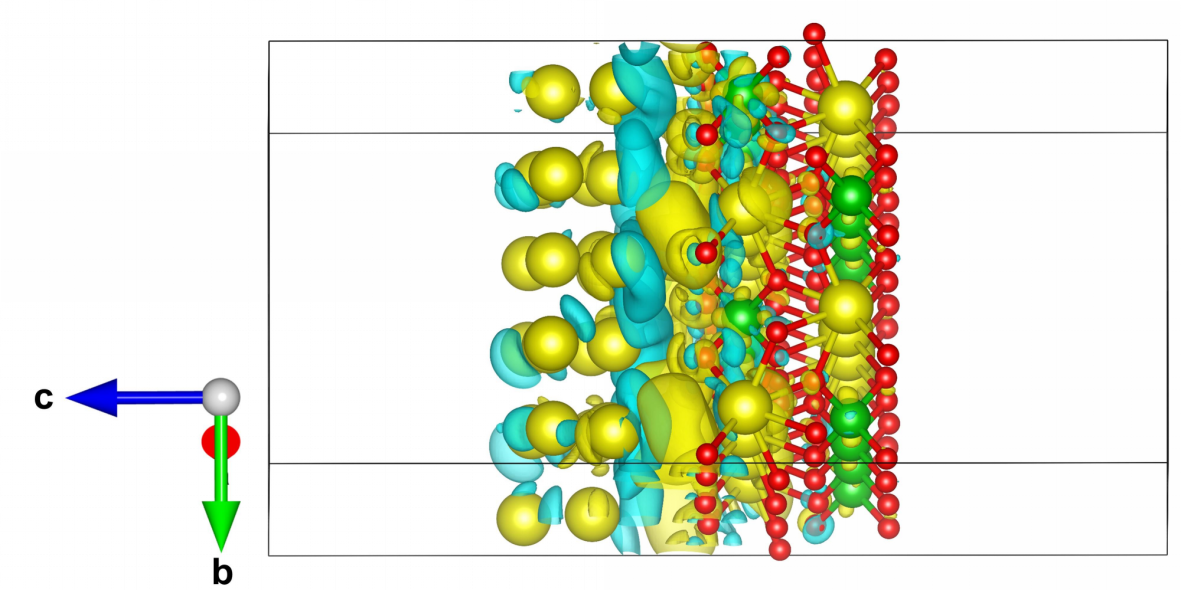


**Figure S30.** Three-dimensional differential charge structure maps corresponding to Fig. 5g_Ⅱ_.

**Table S1.** The cell parameters of BiVO_4_ and Bi/BiVO_4_-450 after XRD refinement.

|  | BiVO_4_ | Bi/BiVO_4_-450 | |
| --- | --- | --- | --- |
|  |  | Bi | BiVO_4_ |
| a (Å) | 5.1985 | 4.5361 | 5.1979 |
| b (Å) | 5.0971 | 4.5361 | 5.0957 |
| c (Å) | 11.7036 | 11.9031 | 11.7011 |
| α (°) | 90 | 90 | 90 |
| β (°) | 90 | 90 | 90 |
| γ (°) | 90.3750 | 90 | 90.3950 |
| V(A^3^) | 310.0180 | 120 | 310.0060 |

**Table S2.** Content of V in BiVO_4_, BiVO_4_-450 Bi/BiVO_4_-450 (after reaction) and Bi/BiVO_4_-500 from ICP test.

| Sample | V (mg/L) |
| --- | --- |
| BiVO_4_ | 1.640 |
| Bi/BiVO_4_-450 | 1.650 |
| Bi/BiVO_4_-450 (After the reaction) | 1.628 |
| Bi/BiVO_4_-500 | 1.450 |

Tab. S3 The percentage content of Bi/BiVO_4_-450 in EDS elemental analysis.

| Elem | Wt. % |
| --- | --- |
| Bi | 50.04 |
| V | 38.49 |
| O | 11.47 |

**Table S4.** Ratios of O_V_ for the BiVO_4_, Bi/BiVO_4_-400, Bi/BiVO_4_-450 and Bi/BiVO_4_-500 derived from XPS spectra.

| Sample | O_V_ (%) |
| --- | --- |
| BiVO_4_ | 15.75 |
| Bi/BiVO_4_-400 | 30.51 |
| Bi/BiVO_4_-450 | 38.51 |
| Bi/BiVO_4_-500 | 30.97 |

**Table S5**. EXAFS fitting parameters at the Bi K-edge for various samples (*Ѕ*_0_^2^=0.88 from Bi_2_O_3_).

| Sample | Shell | *^a^*CN | *^b^*R (Å) | *^c^*σ^2^ (Å^2^) | *^d^*ΔE_0_ (eV) | R factor |
| --- | --- | --- | --- | --- | --- | --- |
| Bi_2_O_3_ | Bi–O | 2 | 2.15 ± 0.01 | 0.0039 | −4.0±2.0 | 0.0041 |
| Bi/BiVO_4_-450 | Bi–O | 2.6 ± 0.2 | 2.20 ± 0.01 | 0.0075 | −4.2±1.7 | 0.0137 |
|  | Bi–V/Bi | 2.9 ± 0.3 | 2.53 ± 0.01 | 0.0150 |  |  |
|  | Bi–Bi | 0.4 ± 0.1 | 3.24 ± 0.01 | 0.0155 |  |  |

^a^CN: coordination numbers; ^b^R: bond distance; ^c^σ^2^: Debye-Waller factors; ^d^ΔE_0_: the inner potential correction. R factor: goodness of fit. Error bounds that characterize the structural parameters obtained by EXAFS spectroscopy were estimated as CN: ±20%; R: ± 1%; σ^2^: ± 20%.

**Table S6**. Comparison of AQY values for photocatalytic oxygen evolution of Bi/BiVO_4_-450 with recently reported photocatalysts.

| Photocatalyst | Light source | AQY (%) | Ref. |
| --- | --- | --- | --- |
| Bi/BiVO_4_-450 | λ=420nm | 29.32 | This work |
| 15% CN/BiVO_4_ | λ=400nm | 2.50 | ^[1]^ |
| O_V_-rich BG | λ=420nm | 23.19 | ^[2]^ |
| BiVO_4_ | λ=420nm | 0.025 | ^[3]^ |
| 30-face BiVO_4_ | λ=430nm | 18.3 | ^[4]^ |
| CZS-BiVO_4_ | λ=420nm | 24.1 | ^[5]^ |
| BiVO_4_ | λ=420nm | 1.88 | ^[6]^ |
| BCO/NCO-30 | λ=420nm | 3.89 | ^[7]^ |
| Au/CoO_X_/BiVO_4_ | λ=420nm | 10.3 | ^[8]^ |
| 2D BiVO_4_ NSs | λ=420nm | 26.1 | ^[9]^ |
| Cu-BiVO_4_@CoO_X_ | λ=420nm | 1.80 | ^[10]^ |

**Table S7.** Fitting parameters of fs-TA decay.

| Sample | τ_1_ | τ_2_ | τ_av_ |
| --- | --- | --- | --- |
| BiVO_4_ | 0.67 | 5.47 | 4.72 |
| Bi/BiVO_4_-450 | 0.96 | 13.05 | 11.85 |

References

[1] Q. Zhang, G. Liu, T. Liu, *ACS Sustain. Chem. Eng.* **2024**, *12*, 5675.

[2] S. Liu, J. Pan, W. Kong, X. Li, J. Zhang, X. Zhang, R. Liu, Y. Li, Y. Zhao, D. Wang, J. Zhang, S. Zhu, *ACS Appl. Mater. Interfaces* **2022**, *14*, 12180.

[3] D. Dai, X. Liang, B. Zhang, Y. Wang, Q. Wu, Z. Wang, Z. Zheng, H. Cheng, Y. Dai, B. Huang, P. Wang, *Adv Sci* **2022**, *9*, 2105299.

[4] P. Li, X. Chen, H. He, X. Zhou, Y. Zhou, Z. Zou, *Adv. Mater.* **2018**, *30*, 1703119.

[5] C. Zeng, Y. Hu, T. Zhang, F. Dong, Y. Zhang, H. Huang, *J. Mater. Chem. A* **2018**, *6*, 16932.

[6] C. Dong, S. Lu, S. Yao, R. Ge, Z. Wang, Z. Wang, P. An, Y. Liu, B. Yang, H. Zhang, *ACS Catal.* **2018**, *8*, 8649.

[7] J. Qu, X. Yang, C. Guo, Y. Cai, Z. Li, J. Hu, C. Ming Li, *J. Colloid Interface Sci.* **2022**, *613*, 265.

[8] Y. Qi, Y. Zhao, Y. Gao, D. Li, Z. Li, F. Zhang, C. Li, *Joule* **2018**, *2*, 2393.

[9] C. Dong, S. Lu, S. Yao, R. Ge, Z. Wang, Z. Wang, P. An, Y. Liu, B. Yang, H. Zhang, *ACS Catal.* **2018**, *8*, 8649.

[10] X. Li, Y. Dong, G. Hu, K. Ma, M. Chen, Y. Ding, *Chem. Asian J.* **2021**, *16*, 2967.
